# Cardiomyocyte microRNA-150 confers cardiac protection and directly represses proapoptotic small proline–rich protein 1A

**DOI:** 10.1172/jci.insight.150405

**Published:** 2021-09-22

**Authors:** Tatsuya Aonuma, Bruno Moukette, Satoshi Kawaguchi, Nipuni P. Barupala, Marisa N. Sepúlveda, Christopher Corr, Yaoliang Tang, Suthat Liangpunsakul, R. Mark Payne, Monte S. Willis, Il-man Kim

**Affiliations:** 1 Department of Anatomy, Cell Biology and Physiology, and; 2 Krannert Institute of Cardiology, Indiana University School of Medicine, Indianapolis, Indiana, USA.; 3 Vascular Biology Center, Medical College of Georgia, Augusta University, Augusta, Georgia, USA.; 4 Division of Gastroenterology and Hepatology,; 5 Wells Center for Pediatric Research, and; 6 Indiana Center for Musculoskeletal Health, Indiana University School of Medicine, Indianapolis, Indiana, USA.

**Keywords:** Cardiology, Cell Biology, G protein&ndash;coupled receptors, Heart failure, Noncoding RNAs

## Abstract

MicroRNA-150 (miR-150) is downregulated in patients with multiple cardiovascular diseases and in diverse mouse models of heart failure (HF). miR-150 is significantly associated with HF severity and outcome in humans. We previously reported that miR-150 is activated by β-blocker carvedilol (Carv) and plays a protective role in the heart using a systemic miR-150 KO mouse model. However, mechanisms that regulate cell-specific miR-150 expression and function in HF are unknown. Here, we demonstrate that potentially novel conditional cardiomyocyte–specific (CM-specific) miR-150 KO (miR-150 cKO) in mice worsens maladaptive cardiac remodeling after myocardial infarction (MI). Genome-wide transcriptomic analysis in miR-150 cKO mouse hearts identifies small proline–rich protein 1a (*Sprr1a*) as a potentially novel target of miR-150. Our studies further reveal that *Sprr1a* expression is upregulated in CMs isolated from ischemic myocardium and subjected to simulated ischemia/reperfusion, while its expression is downregulated in hearts and CMs by Carv. We also show that left ventricular *SPRR1A* is upregulated in patients with HF and that *Sprr1a* knockdown in mice prevents maladaptive post-MI remodeling. Lastly, protective roles of CM miR-150 are, in part, attributed to the direct and functional repression of proapoptotic *Sprr1a*. Our findings suggest a crucial role for the miR-150/SPRR1A axis in regulating CM function post-MI.

## Graphical abstract

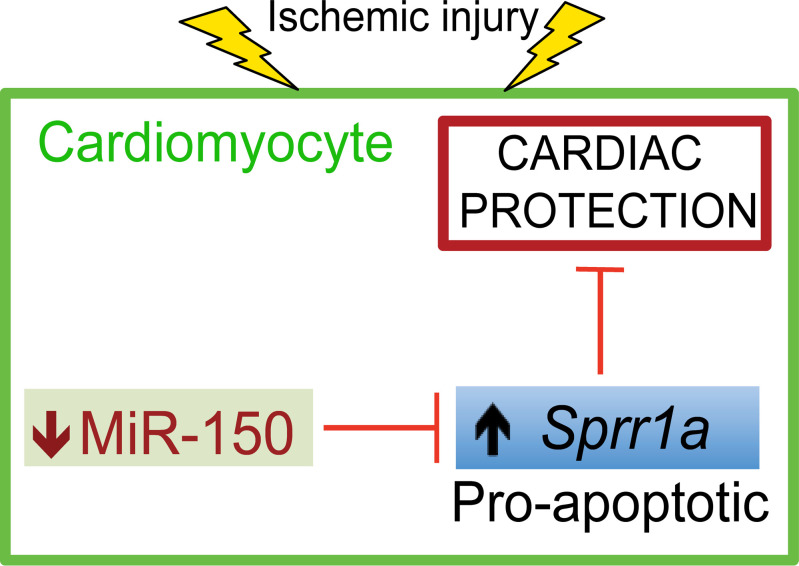

## Introduction

Modulation of microRNA (miR) activity in the heart is an important mechanism that underlies the pathogenesis of heart failure (HF) (1–5). Intriguingly, clinical trials using potentially novel miR therapies are underway for other diseases (6–9) and more recently for HF (e.g., NCT04045405; ClinicalTrials.gov). The circulating or cardiac miR–150-5p (hereafter referred to as miR-150) is downregulated in patients with multiple cardiovascular diseases (CVDs) such as acute myocardial infarction (AMI), atrial fibrillation, dilated cardiomyopathy (DCM), and ischemic cardiomyopathy (10–13), as well as in various mouse models of HF (MI, transverse aortic constriction [TAC], and ischemia/reperfusion [I/R] injury) (14–16). miR-150 is highly conserved and is significantly associated with HF severity and outcome in humans (17). We previously reported that miR-150 is upregulated by the β-blocker carvedilol (Carv) acting through the β-arrestin1–biased β_1_-adrenergic receptor (β_1_AR; receptor found mainly in cardiomyocytes [CMs]) cardioprotective signaling (18). Using a systemic miR-150 KO mouse model, we also showed that β_1_AR/β-arrestin1–responsive miR-150 plays a protective role in the heart (14). Collectively, previous data from both human and rodent studies establish the strong clinical relevance, as well as potential diagnostic, prognostic, and therapeutic applications of miR-150. However, the extent to which expression of miR-150 selectively in CMs regulates MI is unknown, and there is a lack of mechanistic insight by which CM miR-150 modulates cardiac protection.

Small proline–rich protein 1a (SPRR1A) is a stress-inducible protein and is highly conserved in vertebrates. SPRR1A is a substrate of transglutaminase (TGASE) I/II–catalyzed reactions in the formation of the keratinocyte envelope (19). Previous studies linked TGASE II to HF (20, 21) and apoptosis in noncardiac cells (22, 23), suggesting a possible role of SPRR1A as a substrate for TGASE II in the heart. Adenovirus-mediated ectopic overexpression of *Sprr1a* induces the degree of fibrosis, indicated by Masson’s trichrome staining of mouse hearts after TAC (24). In contrast, ectopic overexpression of *Sprr1a* protects CMs and isolated hearts against 2-deoxyglucose and ex vivo I/R (24). Moreover, ectopic *Sprr1a* overexpression does not affect CM survival after reactive oxygen species treatment or serum deprivation (24). This previous report suggests both potential stress/context-dependent effects of *Sprr1a* and the requirement of more physiologically relevant loss-of-function studies to define the exact role of *Sprr1a* in the heart. Interestingly, a previous immunohistochemical study in mouse hearts showed that SPRR1A is upregulated in CMs responding to TAC (24). However, it is unknown whether Carv-responsive miR-150 regulates *Sprr1a* in the heart and whether *Sprr1a* contributes to cardiac pathology.

Using potentially novel mouse models, we demonstrate here that the expression of miR-150 is higher in CMs than other myocardial cells, and conditional CM-specific miR-150 KO (miR-150 cKO) mice enhance cardiac dysfunction, stress, fibrosis, and apoptosis after MI without affecting mortality; transcriptome profiling in miR-150 cKO mice identifies *Sprr1a* as a direct and functional target of CM miR-150; and the hearts of *Sprr1a*-hypomorphic mice are protected against MI. We also show that the expression of *Sprr1a* is upregulated in CMs isolated from ischemic myocardium but is downregulated in hearts and CMs by Carv; cardiac *SPRR1A* is upregulated in patients with HF with reduced ejection fraction (HFrEF), which is inversely associated with the expression of miR-150. Lastly, we demonstrate that miR-150 in CMs plays a protective role, in part, by the direct functional repression of proapoptotic *Sprr1a*. Therefore, the miR-150/SPRR1A dyad may represent a novel therapeutic target for ischemic cardiac injury.

## Results

### CM-specific miR-150 deletion augments cardiac dysfunction and remodeling after MI.

**Figure 1:**
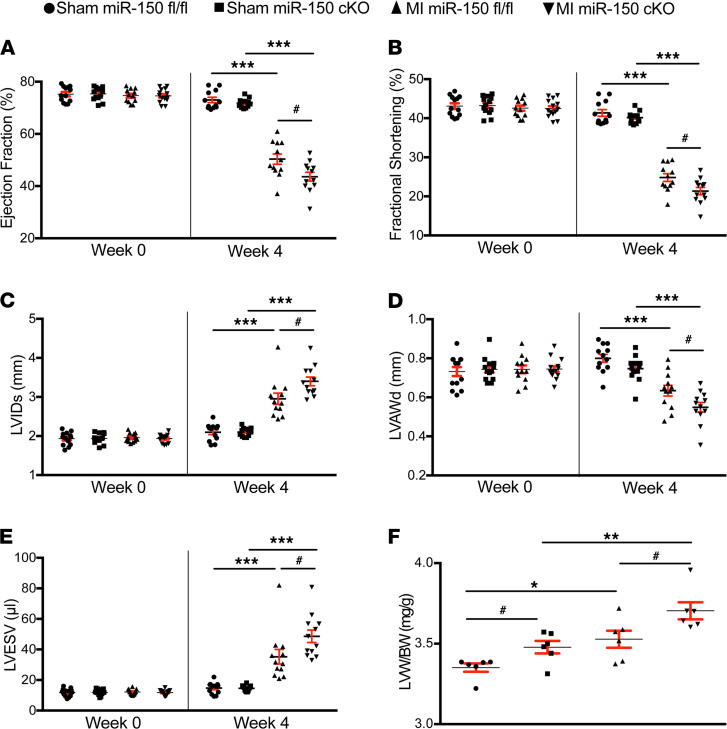
miR-150 selectively in cardiomyocytes protects the mouse heart against MI. (**A–E**) Transthoracic echocardiography was performed at 0 and 4 weeks after MI. Quantification of left ventricular (LV) ejection fraction (EF); fractional shortening (FS); internal diameter, systole (LVIDs); anterior wall thickness, diastole (LVAWd); and end systolic volume (LVESV) is shown. *n* = 12–14 per group. One-way ANOVA with Tukey multiple-comparison test. ***P < 0.001 versus sham; ^#^*P* < 0.05 versus MI miR-150^fl/fl^. (**F**) Left ventricle weight/body weight (LVW/BW) ratio (*n* = 6). One-way ANOVA with Tukey multiple-comparison test. *P < 0.05 or **P < 0.01 versus Sham; ^#^*P* < 0.05 versus miR-150^fl/fl^. Data are presented as mean ± SEM.

To evaluate in vivo roles of CM miR-150 in cardiac stress, we generated a potentially novel miR-150^fl/fl^ mouse line and established CM-specific miR-150 cKO mice by breeding miR-150^fl/fl^ mice with αMHC-Cre mice (Supplemental Figure 1, A–D; supplemental material available online with this article; https://doi.org/10.1172/jci.insight.150405DS1). We first observe that isolated CMs from miR-150 cKO mice have significantly reduced miR-150 levels compared with CMs from miR-150^fl/fl^ mice (Supplemental Figure 2A). However, miR-150 expression in non-CM cell types is comparable between miR-150^fl/fl^ and miR-150 cKO (Supplemental Figure 2, B–D), suggesting CM-specific cKO. Notably, the expression of miR-150 is relatively higher in CMs than other myocardial cells (Supplemental Figure 3). We next show that miR-150 cKO mice have normal cardiac function at baseline (Supplemental Table 2 and Figure 1, A–E).

Despite the normal cardiac function at baseline, miR-150 cKO mice respond differently to ischemic cardiac injury. MI induced by permanent ligation of the left anterior descending (LAD) results in a significant worsening of cardiac function at 3 days, which is indicated by decreased ejection fraction (EF) and fractional shortening (FS) (Supplemental Table 3). miR-150 cKO mice also display impaired cardiac function at 2 weeks after MI, which is shown by a significant decrease in EF, FS, and diastolic anterior wall thickness, as well as a significant increase in end diastolic volume (EDV), end systolic volume (ESV), diastolic interior diameter, and systolic interior diameter (Supplemental Table 4). MI also causes augmented cardiac dysfunction in miR-150 cKO mice at 4 weeks, as evidenced by a significant decrease in EF, FS, and diastolic anterior wall thickness, as well as a significant increase in ESV and systolic interior diameter (Figure 1, A–E, and Supplemental Table 5). In contrast, miR-150^fl/fl^ controls show less functional impairment at 3 days (Supplemental Table 3), 2 weeks (Supplemental Table 4), and 4 weeks following MI (Figure 1, A–E, and Supplemental Table 5). Our morphometric data also show that miR-150 cKO mice have a significant increase in the ratio of left ventricle weight/body weight (LVW/BW) at 4 weeks after sham and MI, when compared with miR-150^fl/fl^ controls (Figure 1F and Supplemental Table 5). However, miR-150 cKO mice do not exhibit an increase in mortality after MI (Supplemental Tables 2–5: see *n* for animal numbers per each group at days 0 and 3, and at weeks 2 and 4 after MI), which was distinct from our systemic miR-150 KO mice with a significant increase in mortality after AMI due to inflammatory response and cardiac rupture (14).

**Figure 2:**
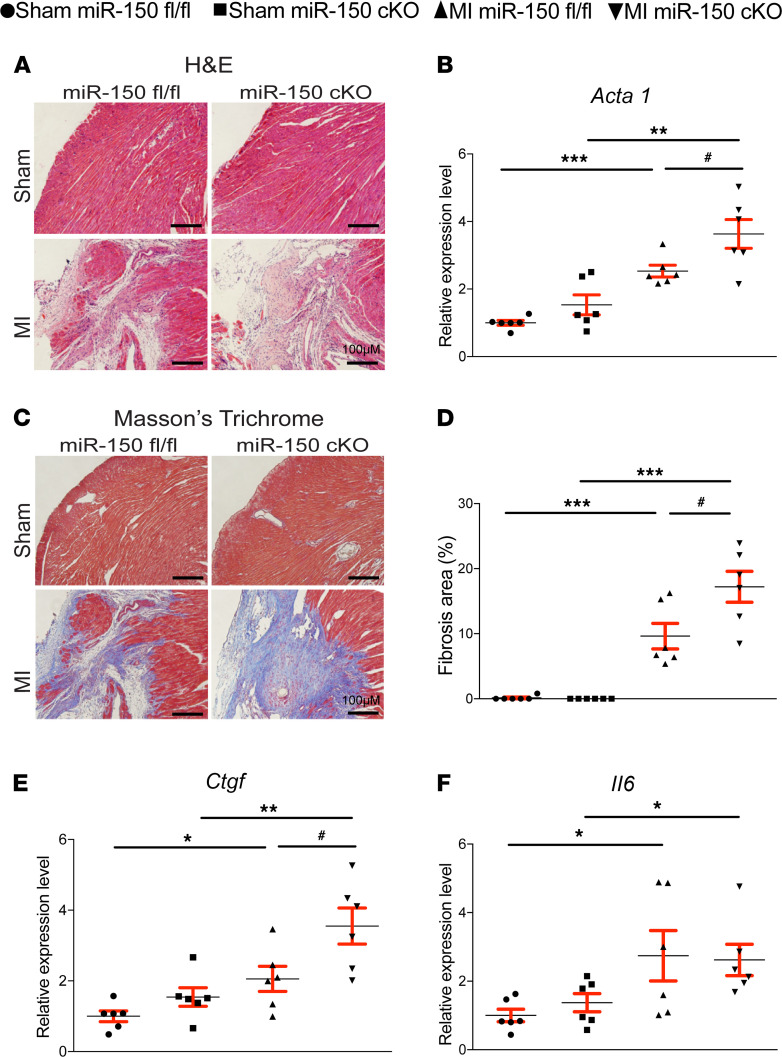
Selective deletion of miR-150 in cardiomyocytes induces cardiac stress and fibrosis after MI. (**A**) Representative H&E staining of heart sections of periischemic border area at 4 weeks after MI shows an increase in loss of normal architecture and cellular integrity, as well as in disorganized structure in miR-150 cKO hearts compared with miR-150^fl/fl^ controls. Scale bars: 100 μm. (**B**) qPCR analysis of *Acta1* expression for cardiac stress in miR-150 cKO hearts compared with miR-150^fl/fl^ controls at 4 weeks after MI. (**C** and **D**) Representative Masson’s trichrome staining (**C**) in heart sections of periischemic border area at 4 weeks after MI and fibrosis quantification in LVs (**D**). Scale bars: 100 μm. (**E** and **F**) qPCR analysis of fibrotic *Ctgf* and inflammatory *Il6* expression in miR-150 cKO hearts compared with miR-150^fl/fl^ controls at 4 weeks after MI. *n* = 6 per group. qPCR data (**B**, **E**, and **F**) are shown as fold induction of gene expression normalized to *Gapdh*. One-way ANOVA with Tukey multiple-comparison test. *P < 0.05, **P < 0.01 or ***P < 0.001 versus sham; ^#^*P* < 0.05 versus MI miR-150^fl/fl^. Data are presented as mean ± SEM.

**Figure 3:**
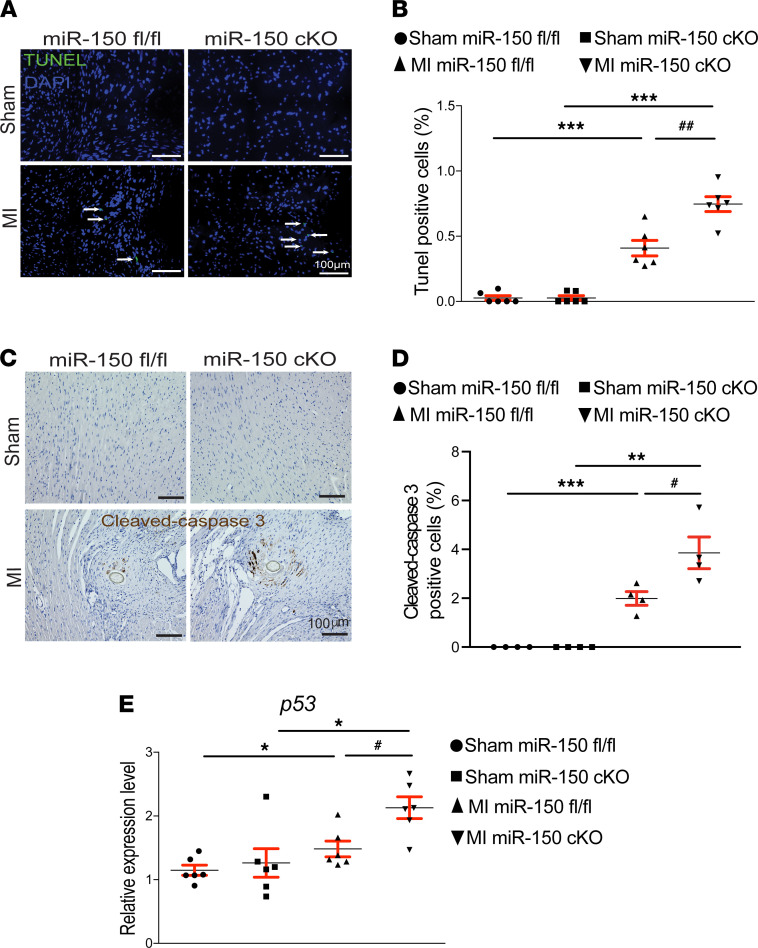
Selective loss of miR-150 in cardiomyocytes induces cardiac apoptosis post-MI. (**A** and **B**) Representative TUNEL staining images in heart sections of periischemic border area at 4 weeks after MI (**A**) and quantification of apoptosis in six 40× fields (**B**). Scale bars: 100 μm. (**C** and **D**) Representative cleaved caspase 3 staining images in heart sections of periischemic border area at 4 weeks after MI (**C**) and quantification of apoptosis in six 40× fields (**D**). Scale bars: 100 μm. (**E**) qPCR analysis of proapoptotic *p53* expression in miR-150 cKO hearts compared with miR-150^fl/fl^ controls at 4 weeks after MI. Data are shown as fold induction of gene expression normalized to *Gapdh*. *n* = 6 per group. One-way ANOVA with Tukey multiple-comparison test. *P < 0.05, **P < 0.01, or ***P < 0.001 versus sham; ^#^*P* < 0.05 or ^##^*P* < 0.01 versus MI miR-150^fl/fl^. Data are presented as mean ± SEM.

We also find that miR-150 cKO hearts exhibit more loss of normal architecture and cellular integrity (Figure 2A), which is consistent with increased mRNA levels of fetal *Acta1* (Figure 2B) after 4 weeks of MI compared with miR-150^fl/fl^ MI hearts. To further investigate the response of miR-150 cKO mice to MI, we assessed the degree of fibrosis using Masson’s trichrome staining of the hearts at 4 weeks after MI. We found small regions of fibrosis in miR-150^fl/fl^ hearts, while miR-150 cKO hearts contained significantly larger fibrotic areas (Figure 2, C and D). Our data further show that miR-150 cKO MI hearts had increased expression of fibrotic *Ctgf* (Figure 2E), as compared with miR-150^fl/fl^ controls. We next examined whether impaired cardiac inflammatory cell response may contribute to the disorganized structure and increased fibrosis in miR-150 cKO MI hearts. Notably, the expression of inflammatory *Il6* and *Tnfa* is comparable between miR-150^fl/fl^ and miR-150 cKO groups (Figure 2F and Supplemental Figure 4). These results were distinct from ones observed in our systemic miR-150 KO mice (14). These data are consistent with no increase in post-MI mortality observed in miR-150 cKO mice (Supplemental Tables 2–5: see *n* for animal numbers per each group from day 0 to week 4 after MI). Lastly, we examined evidence of apoptosis and found that miR-150 cKO MI hearts had significantly elevated numbers of TUNEL^+^ cells (Figure 3, A and B) and cleaved caspase 3^+^ cells (Figure 3, C and D) compared with miR-150^fl/fl^ MI hearts. Our data further show that miR-150 cKO MI hearts had increased mRNA levels of apoptotic *p53* (Figure 3E) compared with miR-150^fl/fl^ MI hearts. Collectively, these results demonstrate that selective knockdown of miR-150 in CMs increases the diverse cardiac structural and functional abnormalities associated with post-MI remodeling.

### CM miR-150 regulates the expression of a relatively small number of cardiac genes after MI involved in adrenergic signaling, DCM, and hypertrophic cardiomyopathy.

To identify potentially novel miR-150 targets, we performed transcriptome profiling of mouse left ventricles (LVs). LV tissues from 8- to 12-week-old miR-150^fl/fl^ and miR-150 cKO mice subjected to sham or MI were interrogated at 4 weeks after MI. Among 24,881 mouse genes that we profiled, 80 genes were significantly upregulated (Supplemental Figure 5A and Supplemental Table 6; see up_Group2_vs_Group1 sheet), and 54 genes were significantly downregulated (Supplemental Figure 5A and Supplemental Table 6; see down_Group2_vs_Group1 sheet) in sham miR-150 cKO compared with sham miR-150^fl/fl^. We also find that 104 genes are significantly upregulated (Supplemental Figure 5B and Supplemental Table 6; see up_Group3_vs_Group1 sheet), and 35 genes were significantly downregulated (Supplemental Figure 5B and Supplemental Table 6; see down_Group3_vs_Group1 sheet) in MI miR-150^fl/fl^ compared with sham miR-150^fl/fl^. In addition, 335 genes were significantly upregulated, and 397 genes were significantly downregulated (Supplemental Figure 5C) in MI miR-150 cKO compared with sham miR-150 cKO. Lastly, we observed that 427 genes were significantly upregulated (Supplemental Figure 5D and Supplemental Table 6; see up_Group4_vs_Group3 sheet), and 265 genes were significantly downregulated (Supplemental Figure 5D and Supplemental Table 6; see down_Group4_vs_Group3 sheet) in MI miR-150 cKO compared with MI miR-150^fl/fl^.

To identify the functional roles of differentially regulated genes observed in miR-150 cKO, we then classified differentially expressed genes by the Kyoto Encyclopedia of Genes and Genomes (KEGG) signaling pathway classification system. Our signaling pathway analysis in sham miR-150 cKO compared with sham miR-150^fl/fl^ demonstrates that upregulated genes are related to Relaxin signaling pathway and extracellular matrix–receptor (ECM-receptor) interaction (Supplemental Figure 6A). We also found that the top canonical signaling pathways for upregulated genes in MI miR-150^fl/fl^ compared with sham miR-150^fl/fl^ included ECM-receptor interaction, focal adhesion, and Relaxin signaling pathway (Supplemental Figure 6B). In contrast, downregulated genes were involved in signaling pathways regulating pluripotency of stem cells, the mTOR signaling pathway, the Wnt signaling pathway, and the Hippo signaling pathway (Supplemental Figure 7A). Moreover, the expression of genes involved in protein digestion and absorption, the PI3K-Akt signaling pathway, and the MAPK signaling pathway were significantly upregulated (Supplemental Figure 6C), while the expression of genes involved in ribosome, oxidative phosphorylation, and thermogenesis were significantly downregulated (Supplemental Figure 7B) in MI miR-150 cKO compared with sham miR-150 cKO. Lastly, we observed that genes involved in adrenergic signaling in CMs, DCM, hypertrophic cardiomyopathy (HCM), and oxytocin signaling pathway were significantly increased (Supplemental Figure 6D) in MI miR-150 cKO compared with MI miR-150^fl/fl^. In contrast, genes involved in ribosome, oxidative phosphorylation, and HCM were significantly decreased (Supplemental Figure 7C). Overall, our transcriptomic data indicate that miR-150 in CMs modulated the expression of a subset of genes/signaling pathways, which regulated cardiac function and structure.

### CM miR-150 directly represses a novel target, Sprr1a.

**Figure 4:**
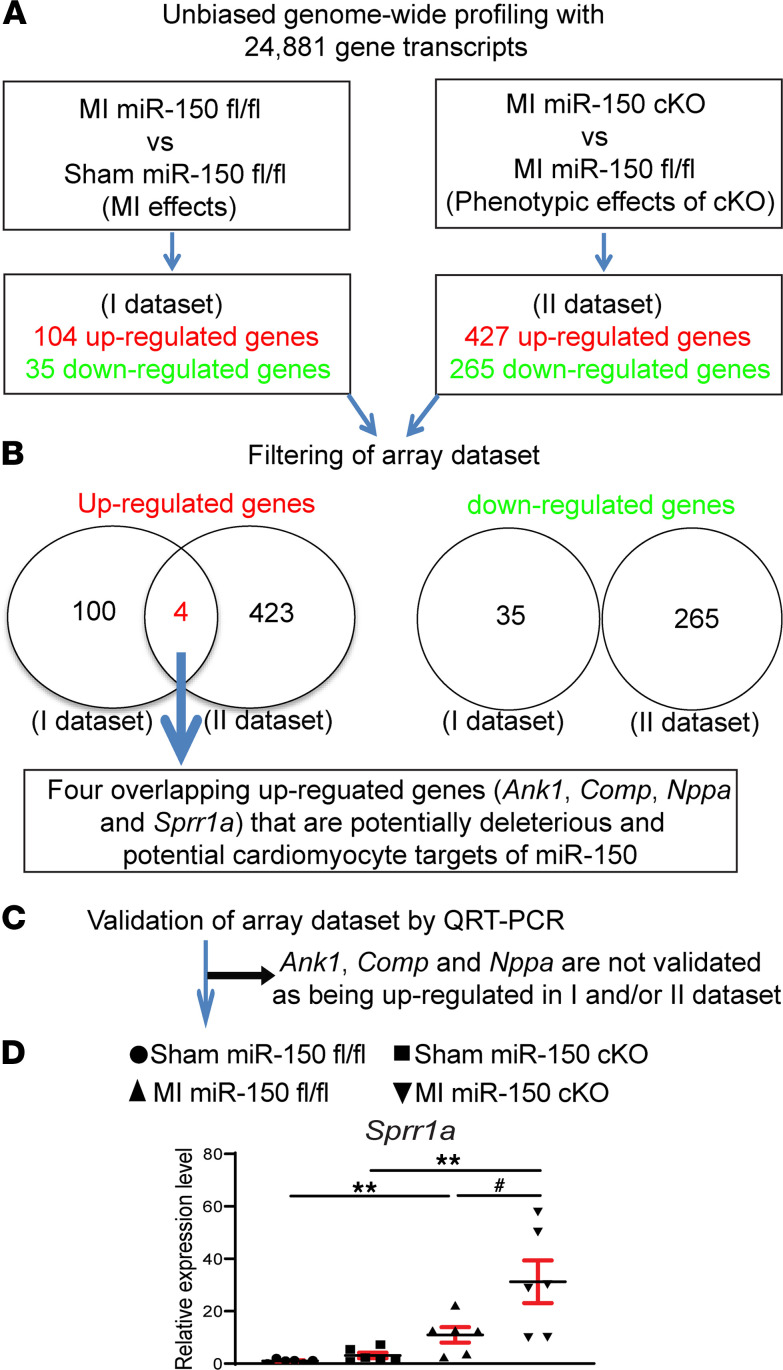
Transcriptome profiling in miR-150 cKO mice identifies *Sprr1a* as a target of miR-150. (**A** and **B**) Transcriptome profiling (**A**) and filtering strategy of array data set based on the correlation between cardiac phenotypes and transcript signatures (**B**). Four dysregulated (DE) genes, which are upregulated in both the I data set (MI miR-150^fl/fl^ compared with sham miR-150^fl/fl^ controls) and the II data set (MI miR-150 cKO compared with MI miR-150^fl/fl^) at 4 weeks after MI, were chosen for further analyses. Note that there are no overlapping downregulated genes in I data set and II data set. *n* = 3 per group. (**C** and **D**) Validation strategy of array data set. Four DE genes (*Ank1*, *Comp*, *Nppa*, and *Sprr1a*) were validated by qPCR analyses of potentially deleterious genes in hearts from miR-150^fl/fl^ and miR-150 cKO mice at 4 weeks after MI. Note that *Ank1*, *Comp*, and *Nppa* are not validated to be upregulated in I data set and/or II data set by qPCR analyses. Data are shown as fold induction of gene expression normalized to *Gapdh*. *n* = 6 per group. One-way ANOVA with Tukey multiple-comparison test. **P < 0.05 versus sham; ^#^*P* < 0.05 versus MI miR-150^fl/fl^. Data are presented as mean ± SEM.

To discover novel miR-150 targets that control cardiac pathophysiology and impair CM responses, we filtered 4 significantly dysregulated genes from our array data set based on the correlation between cardiac phenotypes and transcript signatures from MI miR-150^fl/fl^ versus Sham miR-150^fl/fl^, or MI miR-150 cKO versus MI miR-150^fl/fl^ (Figure 4, A and B). The rationale to focus on these 4 potentially maladaptive genes (*Ank1*, *Comp*, *Nppa* and *Sprr1a*) is that they are upregulated in both MI miR-150^fl/fl^ versus Sham miR-150^fl/fl^ (i.e., MI effects) and MI miR-150 cKO versus MI miR-150^fl/fl^ (i.e., phenotypic effects of cKO). Using real-time PCR analyses, we validated among the 4 genes that LV *Sprr1a* (not *Ank1*, *Comp*, or *Nppa*) was upregulated after MI and in MI miR-150 cKO mice compared with MI miR-150^fl/fl^ controls (Figure 4, C and D). Notably, we demonstrated that *Sprr1a* expression was upregulated in post-MI hearts (Figure 4D), concurrent with cardiac miR-150 downregulation (14). Our transcript profiling and validation analyses suggest that *Sprr1a* is a novel target of CM miR-150 after MI.

**Figure 5:**
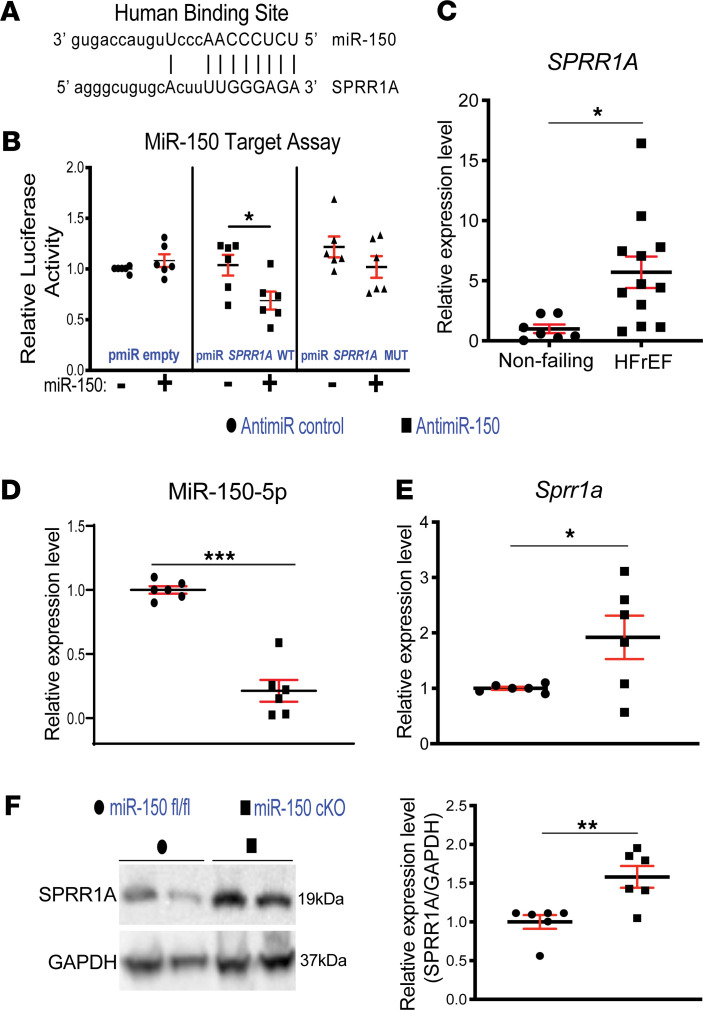
*SPRR1A* is a direct target of miR-150 and is upregulated in patients with heart failure. (**A**) Human *SPRR1A* has a miR-150 binding site. miR-150 seed pairing in the target region is presented as vertical lines. (**B**) The ability of miR-150 to directly inhibit the activity of luciferase (LUC) reporter constructs that contain either WT or mutated (MUT) binding site for *SPRR1A*. Transfection with or without miR-150 mimic in H9c2 cells is shown. Firefly LUC activity was normalized to Renilla LUC activity and compared with empty vector measurements. Results are representative of 6 independent experiments with different biological samples. Unpaired 2-tailed *t* test. *P < 0.05 versus miR mimic control. (**C**) qPCR expression analysis of *SPRR1A* in heart tissues from patients with heart failure with reduced ejection fraction (HFrEF; *n* = 12) relative to nonfailing heart tissues (*n* = 7). Data are shown as fold change of gene expression normalized to *GAPDH*. Unpaired 2-tailed *t* test. *P < 0.05 versus nonfailing. (**D** and **E**) RNAs isolated from H9c2 cells transfected with 100 nM MirVana miR-150 inhibitor or 15-mer control were analyzed by miR-150–specific qPCR to access the levels of miR-150 (**D**). Levels of *Sprr1a* are indicated in **E**. Data were normalized to U6 snRNA (**D**) or *Gapdh* (**E**) and expressed relative to antimiR control. Results are from 6 independent experiments with different biological samples. Unpaired 2-tailed *t* test. *P < 0.05 or ***P < 0.001 versus antimiR control. (**F**) SPRR1A protein levels were measured in whole heart lysates from miR-150 cKO mice compared with miR-150^fl/fl^. Results are from 6 independent experiments with different biological samples. Unpaired 2-tailed *t* test. **P < 0.01 versus miR-150^fl/fl^. Data are presented as mean ± SEM.

We next employed bioinformatic miR target prediction tools (25–28) and identified a putative binding site for miR-150 in human *SPRR1A*. Interestingly, rodent and human *Sprr1a* genes have almost identical genomic organization and exon/intron sizes (29) and have 1 miR-150 binding site, suggesting the evolutionary conservation of miR-150’s regulation of *Sprr1a* and their roles. To examine if *Sprr1a* is a direct target of miR-150 repression, we transfected CMs with constitutively active luciferase (LUC) reporter constructs containing the binding site of miR-150 in human *SPRR1A* (Figure 5A) and miR-150 mimics. We observed the repression of LUC activity by miR-150 for the WT *SPRR1A* reporter. When we mutated seed binding sites for miR-150, the LUC reporter was insensitive to miR-150 overexpression (Figure 5B), suggesting the specific dependence of target sites on miR-150. Thus, our results strongly indicate that *Sprr1a* is a novel direct target of miR-150.

SPPR1A is a substrate of TGASE II. TGASE II is linked to HF (20, 21) and apoptosis of noncardiac cells (22, 23), suggesting that SPRR1A may play a role in the heart and apoptosis. Interestingly, the protein level of SPPR1A was reported to be increased in CMs after TAC (24). Consistent with this previous report, we show that *Sprr1a* was upregulated in CMs isolated from ischemic hearts at 7 days after MI (Supplemental Figure 8). Our fractionation studies of different cardiac cell types further show that *Sprr1a* is also upregulated in CFs isolated from mouse hearts after MI (Supplemental Figure 8). We also found that LV *SPRR1A* is upregulated in patients with HFrEF (Figure 5C), concurrent with downregulation of cardiac miR-150 (10, 11). Our human data on *SPRR1A* are consistent with isoproterenol-induced (ISO-induced) myocardial injury (30) and renal I/R injury in mice (31), as well as is in agreement with our mouse data (Figure 4D).

Given that *Sprr1a* expression is upregulated in CMs isolated from ischemic myocardium (Supplemental Figure 8) and that CM miR-150 is an important regulator of MI (Figure 1, Figure 2, and Figure 3), we performed loss-of-function studies in CMs. We found that *Sprr1a* is upregulated by miR-150 inhibition (Figure 5, D and E). This CM result is confirmed by our in vivo protein analysis, revealing significantly elevated levels of SPRR1A in miR-150 cKO mouse hearts compared with miR-150^fl/fl^ controls (Figure 5F). Thus, our data suggest that *Sprr1a* is a key direct target of miR-150 in CMs and hearts.

### Hypomorphic mutation of Sprr1a in mice prevents maladaptive post-MI remodeling.

**Figure 6:**
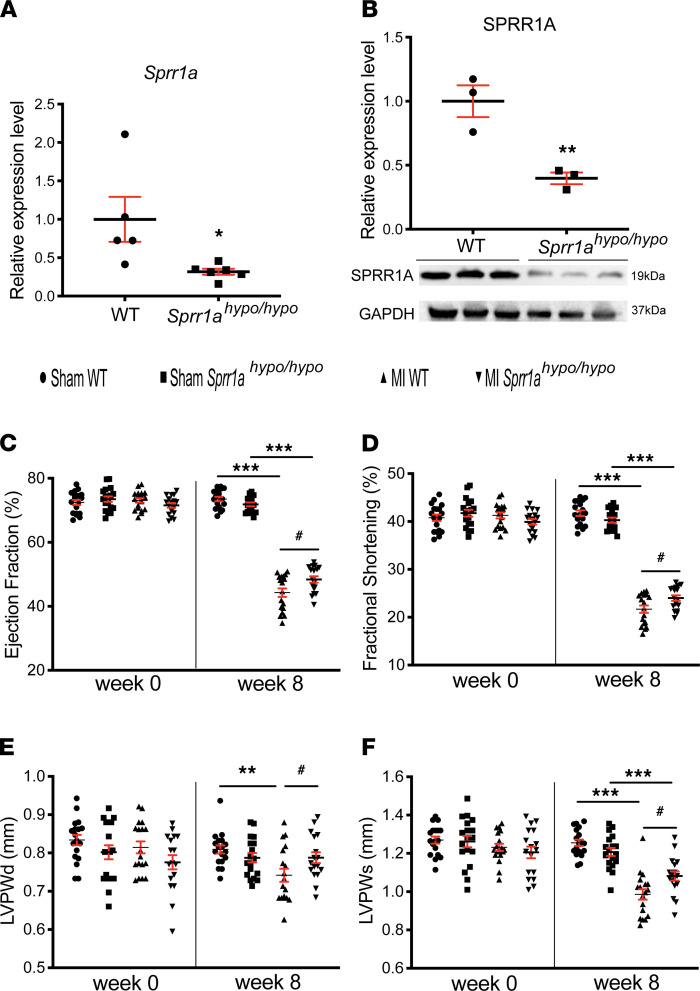
Knockdown of *Sprr1a* protects the mouse heart against MI. (**A**) qPCR expression analysis of *Sprr1a* in heart tissues from *Sprr1a^hypo/hypo^* mice (*n* = 6) relative to WT controls (*n* = 5). Data are shown as fold change of gene expression normalized to *Gapdh*. Unpaired 2-tailed *t* test. *P < 0.05 versus WT. (**B**) SPRR1A protein levels were measured in whole heart lysates from *Sprr1a^hypo/hypo^* mice compared with WT. Results are from 3 different biological samples. Unpaired 2-tailed *t* test. **P < 0.01 versus WT. (**C–F**) Transthoracic echocardiography was performed at 0 and 8 weeks after MI. Quantification of LV ejection fraction (**C**); fractional shortening (**D**); posterior wall thickness, diastole (LVPWd) (**E**); and posterior wall thickness, systole (LVPWs) (**F**). *n* = 17–18 per group. One-way ANOVA with Tukey multiple-comparison test. **P < 0.01 or ***P < 0.001 versus Sham; ^#^*P* < 0.05 versus MI WT. Data are presented as mean ± SEM.

To evaluate in vivo roles of *Sprr1a* in cardiac stress, we obtained a potentially novel *Sprr1a*-hypomorphic mouse line. We first observed that *Sprr1a*-hypomorphic (*Sprr1a^hypo/hypo^*) mouse hearts had significantly reduced *Sprr1a* and SPRR1A levels compared with WT mice (Figure 6, A and B). We next show that *Sprr1a*-hypomorphic mice had normal cardiac function at baseline (Supplemental Table 7 and Figure 6, C–F). However, *Sprr1a*-hypomorphic mutation subjected to MI resulted in a significant improvement of cardiac function at 4 weeks, which is indicated by an increase in FS and cardiac output (CO) (Supplemental Table 8). *Sprr1a^hypo/hypo^* mice also displayed enhanced cardiac function at 8 weeks after MI, as evidenced by a significant increase in EF, FS, diastolic posterior wall thickness, and systolic posterior wall thickness (Figure 6, C–F, and Supplemental Table 9) as compared with WT controls.

**Figure 7:**
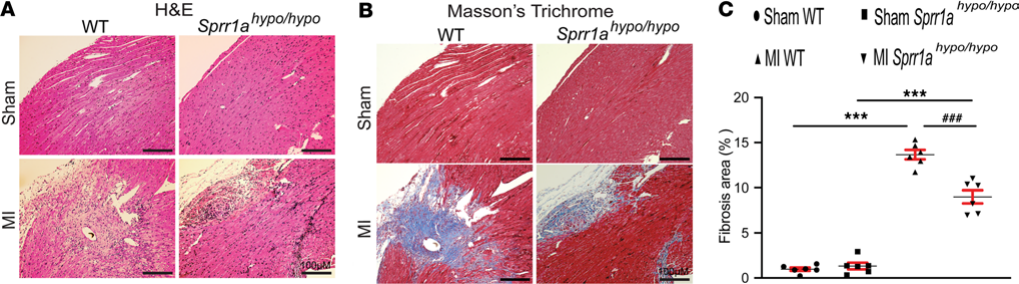
Downregulation of *Sprr1a* in mice reduces cardiac stress and fibrosis after MI. (**A**) Representative H&E staining of heart sections of periischemic border area at 8 weeks after MI shows a decrease in disorganized structure, as well as in loss of normal architecture and cellular integrity in *Sprr1a^hypo/hypo^* hearts compared with WT controls. Scale bars: 100 μm. (**B** and **C**) Representative Masson’s trichrome staining in heart sections of periischemic border area at 8 weeks after MI (**B**) and fibrosis quantification in LVs (**C**). Scale bars: 100 μm. One-way ANOVA with Tukey multiple-comparison test. ***P < 0.001 versus sham; ^###^*P* < 0.001 versus MI WT. Data are presented as mean ± SEM.

We also found that *Sprr1a*-hypomorphic mouse hearts exhibited less loss of normal architecture and cellular integrity (Figure 7A) after 8 weeks of MI compared with WT MI hearts. We assessed the degree of fibrosis using Masson’s trichrome staining of the hearts at 8 weeks after MI. We observed that *Sprr1a*-hypomorphic mouse hearts contained significantly smaller fibrotic areas than WT hearts after MI (Figure 7, B and C). In agreement with our histology data, *Sprr1a*-hypomorphic MI hearts also exhibited decreased expression of inflammatory *Ptprc* and fibrotic *Col1a1* (Supplemental Figure 9), as compared with WT controls. Taken together, these results demonstrate that knockdown of *Sprr1a* in mice improves diverse abnormalities during postischemic cardiac structural/functional remodeling.

### miR-150 functions as a gatekeeper of CM survival, in part, by inhibiting proapoptotic Sprr1a.

Given that miR-150 is upregulated in mouse hearts by Carv (18) but is downregulated in CMs exposed to low oxygen conditions (14), we next tested whether the potentially novel target of miR-150, *Sprr1a*, is inversely regulated in hearts of WT mice or CMs treated with Carv, as well as in CMs subjected to simulated I/R (sI/R) conditions. Indeed, *Sprr1a* was downregulated in mouse hearts after Carv (Supplemental Figure 10A) and in mouse and rat CMs subjected to sI/R conditions after Carv (Supplemental Figure 10, B and C). We also observed that *Sprr1a* was upregulated in CMs after sI/R (Supplemental Figure 10, B and C), which is consistent with our in vivo results after MI (Figure 4D). These results strongly suggest that *Sprr1a* is a critical functional target of CM miR-150 because miR-150 is downregulated in sI/R and MI (14), as well as I/R (32, 33).

**Figure 8:**
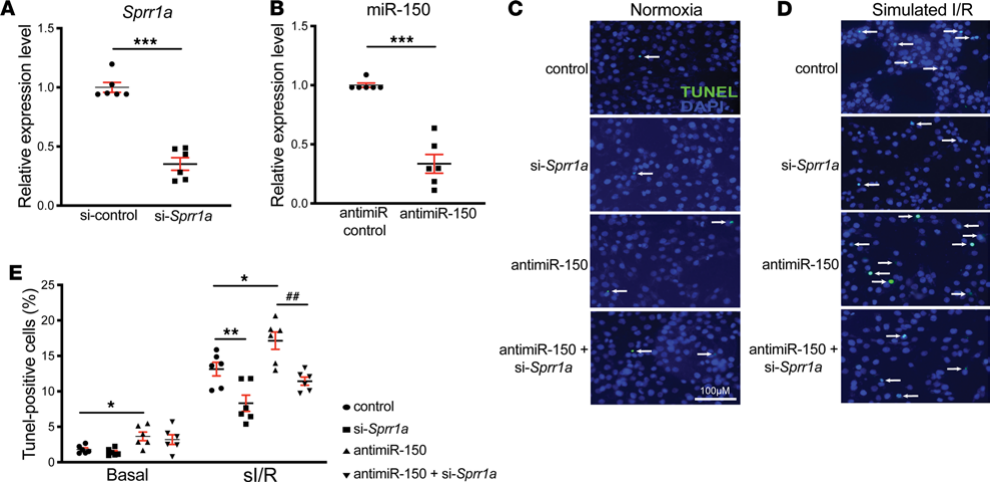
*Sprr1a* is necessary for miR-150–dependent regulation of cardiomyocyte apoptosis. (**A** and **B**) H9c2 cells were transfected with control scramble siRNA (si-control) or *Sprr1a* siRNA (si-*Sprr1a*) (**A**) and with antimiR control scramble or antimiR-150 (**B**). qPCR for *Sprr1a* (**A**) or miR-150 (**B**) were performed to check the knockdown efficiency. *n* = 6 per group. Unpaired 2-tailed *t* test. ***P < 0.001 versus si-control or anti-miR control. (**C–E**) RNA interference with *Sprr1a* protects CMs from the proapoptotic effects of anti–miR-150. CMs were subjected to in vitro simulation of I/R (sI/R). TUNEL assays were then performed in both normoxic (**C** and **E**) and sI/R conditions (**D** and **E**). The percentage of apoptotic nuclei (green) was calculated by normalizing total nuclei (blue). The quantification of data is from 6 independently obtained biological samples. One-way ANOVA with Tukey multiple-comparison test. *P < 0.05 or **P < 0.01 versus control: either si-control or antimiR control. ^##^*P* < 0.01 versus antimiR-150. Data are presented as mean ± SEM.

We next investigated if the target of miR-150, *Sprr1a*, regulates CM apoptosis. We first show that knockdown of *Sprr1a* in CMs decreased protein levels of EGR2 (Figure 8A and Supplemental Figure 11A), which is known to mediate CM apoptosis (14). In agreement with this in vitro data, we also found that, compared with WT controls, *Sprr1a*-hypomorphic hearts exhibited decreased expression of proapoptotic *P2x7r* (Supplemental Figure 11B), which is a direct and functional target of miR-150 in CM apoptosis (14). Interestingly, *P2x7r* expression is increased in miR-150 cKO hearts (Supplemental Figure 11C), concurrent with downregulation of *P2x7r* in *Sprr1a*-hypomorphic hearts. Our further loss-of-function approaches uncover that, compared with controls, *Sprr1a* knockdown (Figure 8A and Supplemental Figure 12A) decreased CM apoptosis in response to sI/R (Figure 8, C–E). This result indicates that *Sprr1a* is sufficient to increase sI/R-mediated apoptosis in CMs. To establish a functional relationship between miR-150 and *Sprr1a* in CM apoptosis, we then applied an antimiR/siRNA-based rescue strategy to validate the functional relevance of *Sprr1a*. Anti–miR-150 treatment induced protein levels of SPRR1A (Supplemental Figure 12A) consistent with quantitative PCR (qPCR) data (Figure 5, D and E). miR-150 knockdown also increased protein levels of proapoptotic SPRR1A and CM apoptosis, which are attenuated by siRNA against *Sprr1a* (Figure 8, B–E, and Supplemental Figure 12A). These TUNEL and Western blot data are consistent with our qPCR data, showing that *Sprr1a* knockdown decreases the expression of *Ing4* and *Klf13* that are known to induce CM apoptosis (34, 35) (Supplemental Figure 12, B and C). We also show that miR-150 knockdown increased the expression of *Ing4* and *Klf13*, which is attenuated by knockdown of *Sprr1a* (Supplemental Figure 12, B and C). Taken together, these CM results, along with our in vivo evidence, suggest that CM miR-150 elicits protective effects in part by the direct functional inhibition of proapoptotic *Sprr1a*.

## Discussion

Here, we identify miR-150 selectively in CMs as a crucial ischemic injury-responsive protector against cardiac and CM apoptosis. Mice selectively deficient for miR-150 in CMs are sensitized to MI, as evident by elevated cardiac apoptosis and fibrosis, as well as impairment of ventricular function. Mechanistically, we identified that miR-150 directly inhibits proapoptotic *Sprr1a* such that the increased expression of *Sprr1a* in CM-specific miR-150 cKO mice or that CMs lacking miR-150 result in a higher degree of sustained CM death during ischemic stress.

The β_1_AR is expressed primarily in the heart (mainly in CMs), and β-arrestin–mediated β_1_AR signaling elicits cardioprotective effects by promoting CM survival after injury (36). We previously reported that miR-150 is activated by Carv-mediated β_1_AR/β-arrestin1 signaling (18). Together with our data presented here, we postulate that CM miR-150 may represent a downstream mechanism by which β_1_AR-mediated β-arrestin signaling pathways confer cardiac protection, and that β-arrestin1–biased β_1_AR regulatory mechanism of miR-150 activation in CMs elicits beneficial remodeling in failing hearts by repressing CM apoptosis through regulation of proapoptotic genes such as *Sprr1a*. Interestingly, we previously reported that systemic miR-150 deletion leads to maladaptive cardiac remodeling by increasing cell death without affecting post-MI neovascularization (14), whereas another group showed that systemic overexpression of miR-150 in mice via AgomiR protects the heart against AMI by decreasing monocyte migration (15). Moreover, cardiac-specific genetic overexpression of miR-150 attenuates TAC-induced cardiac hypertrophy and dysfunction (16). Although these previous studies have shown the importance of miR-150 in HF, our overall knowledge of its actions remains in its infancy, in part due to (a) the lack of mechanistic insight by which CM miR-150 regulates cardiac protection and (b) the absence of definitive and rigorous studies using appropriate mouse models to address the issue of their cardiac versus extra-cardiac actions. In this study, we make discoveries to define the in vivo role of miR-150 selectively in CMs, as well as to identify its potentially novel direct and functional target, *Sprr1a*.

By using transcriptomic profiling approaches, we also identified that CM miR-150 regulates the expression of a relatively small number of cardiac genes after MI. These genes are enriched in known and unknown pathways for miR-150, including adrenergic signaling in CMs, DCM, and HCM. Although additional studies will be warranted to validate the relationship between miR-150’s cardioprotective actions in CMs and the individual genes/pathways identified in our study, our current filtering and validation analyses show that *Sprr1a* was upregulated in the LV tissues of MI miR-150^fl/fl^ when compared with the sham miR-150^fl/fl^ controls, as well as in the LV tissues of MI miR-150 cKO when compared with the MI miR-150^fl/fl^ controls. This led us to identify *Sprr1a* as a potentially novel target of CM miR-150 and suggests that miR-150’s protective actions may be, in part, due to the direct repression of *Sprr1a* expression.

SPRR1A is a substrate of TGASE II that is linked to HF (20, 21) and apoptosis of noncardiac cells (22, 23), suggesting a possible role of *Sprr1a* in the heart. In agreement with this notion, our data show that CMs with knockdown of *Sprr1a* were protected against apoptosis in sI/R (Figure 8, D and E). Interestingly, *Sprr1a* expression is increased in post-MI hearts (24), concurrent with miR-150 downregulation (14). SPRR1A is also upregulated in CMs from TAC-induced myocardium (24). Our cardiac cell fractionation data likewise show that *Sprr1a* was upregulated in CMs isolated from mouse hearts after MI (Supplemental Figure 8). Notably, we also show that *Sprr1a* was upregulated in CFs isolated from post-MI hearts (Supplemental Figure 8), suggesting a potential role of *Sprr1a* in CFs. In the current study, we also demonstrate that LV *SPRR1A* was upregulated in patients with HFrEF (Figure 5C), which is consistent with reports in mice with ISO-induced myocardial injury (30) and renal I/R injury (31). Interestingly, *SPRR1A* expression was also reported to be inversely linked with survival of cancer patients (37–39). Here, we also show that *Sprr1a* was downregulated in hearts and CMs by Carv (Supplemental Figure 10), concurrent with upregulation of miR-150 (18). Consistent with our findings, the cardiac upregulation of *Sprr1a* after cardiac injury is attenuated by treatment with another cardioprotective agent, Danshen (30). Notably, we demonstrate that *Sprr1a* knockdown in mice protected hearts against MI (Figure 6 and Figure 7). Thus, the findings of upregulation of *Sprr1a* in CMs during MI support that repression of this gene could be therapeutically beneficial for HF. Given our results that proapoptotic *Sprr1a* is a direct and functional target for CM miR-150 (Figure 4, Figure 5, and Figure 8), MI patients with decreased levels of miR-150 could be, in particular, considered for future targeted treatments based on *Sprr1a*.

In conclusion, our results using potentially novel loss-of-function mouse models indicate that miR-150 selectively in CMs or *Sprr1a* knockdown in mice protects the heart from ischemic injury and that CM miR-150 directly and functionally represses proapoptotic *Sprr1a*. Interestingly, a previous study reported that systemic overexpression of miR-150 protects the mouse heart against AMI by repressing monocyte migration and CXCR4 (15). Although this non–cell type–specific study using miR-150 mimics indicates a role for miR-150 in post-AMI monocyte recruitment (i.e., the extracardiac action of miR-150), our CM-specific cKO studies clearly define the cardiac actions of miR-150 in MI and identify the underlying mechanism by which miR-150 in CMs selectively affects myocardium apoptosis. Given that downregulation of miR-150 also underlies cardiac hypertrophy and other forms of cardiac disease (11, 32, 33, 40), the protective role of miR-150 in CMs may be commonly applied to multiple stress settings. Thus, boosting miR-150 levels via Carv or miR-150 overexpression could be an attractive adjunctive strategy to provide therapeutic benefits, in part, by attenuating CM apoptosis.

### Study limitation.

Although we demonstrate that miR-150 expression in CMs is an essential regulator of MI, it is possible that expression in other myocardial cells also plays a prominent role indicated by our data (Supplemental Figure 3A). Future studies using our miR-150^fl/fl^ mouse line and other cell-specific Cre mice are, thus, warranted to fully understand the possible contribution of miR-150 expression in other cell types to postischemic heart remodeling and the detailed underlying mechanisms of extracardiac actions (e.g., inflammatory response) mediated by miR-150. miR-150 may also have other targets mediating distinct functions. We will investigate additional functional targets (i.e., *Sprr1a*-independent mechanisms) by cross-referencing the gene signature from miR-150 cKO mice (Supplemental Figure 5 and Supplemental Table 6) with prediction analyses of miR-150 binding sites in our future mechanistic studies. Notably, downstream mechanisms of *Sprr1a* to regulate CM apoptosis remain elusive, although our current data (Supplemental Figures 11 and 12) suggest that *Sprr1a* activates proapoptotic markers. Whether *Sprr1a* is functionally regulated by miR-150 in non-CM cell types and hearts is also unknown and beyond the scope of the current investigation. Moreover, additional histopathological and IHC analyses at earlier time points after MI are needed to get a much more complete understanding of the sequence of events. To measure cardiac function more accurately and assess diastolic dysfunction, additional methods such as Simpson method (biplane method of disks: a modality requiring area tracings of LV cavity) with multiple B-mode images and pressure-volume loop analysis would be required. Lastly, other in vivo injury models (e.g., I/R), as well as detailed studies on other roles of the miR-150/SPRR1A dyad in CMs and non-CM cell types, are warranted before pursuing this axis as a vital therapeutic approach.

## Methods

### Availability of data and materials.

The microarray data discussed in this publication have been deposited in NCBI’s Gene Expression Omnibus and are accessible through GEO Series access number GSE177057 (https://www.ncbi.nlm.nih.gov/geo/query/acc.cgi?acc=GSE177057). Supplemental Methods are available online with this article.

### Cardiac-specific KO of miR-150 in mice.

To establish CM-specific miR-150 cKO mice, we generated a miR-150^fl/fl^ mouse line (Cyagen US Inc., NTMCK-160902-ACD-01) using the CRISPR/Cas9 strategy as shown in Supplemental Figure 1A. In brief, mouse genomic fragments containing homology arms and the cKO region were amplified from a BAC clone by using high-fidelity Taq DNA Polymerase (New England Biolabs). The fragments were sequentially assembled into a targeting vector together with recombination sites and selection markers. The final targeting vector was digested by appropriate restriction enzymes and was sequenced for confirmation purposes. The rigorous off-target prevention was performed using the whole-genome off-target risk calculation.

The guide RNA to mouse miR-150 gene, the donor DNA containing LoxP sites, and the Cas9 mRNA were coinjected into fertilized mouse eggs to generate targeted knockin offspring. The founder (F0) animals were identified by PCR, followed by sequence analysis, which were then bred to WT mice to test germline transmission and to get the first generation (F1) animals,, as well as to eliminate any potential off-target effect. The genotyping strategy is shown in Supplemental Figure 1B and the following PCR primers were used to screen positive F1 mice. For region 1, forward: 5′-CTCGTGAATGCTGGATCAAAGGTG-3′ and reverse: 5′-GTACTGTGGATTCGGACCAGTCTG-3′. For region 2, forward: 5′-ACGTAAACGGCCACAAGTTCAGATC-3′ and reverse: 5′-TCCTCGTTGTCTTACGCATTAGCTG-3′. The genotyping result of this long-PCR method was confirmed by the short-PCR method described below to exclude any potential random inserts in homologous recombinant F0 mice.

All F1 mice were also confirmed by sequencing using the following primers on the homology arm and cKO region. For region 1: 5′-TGCTAGGCTCTCAGGCAGTGTTC-3′. For region 2: 5′-CAGCAGCAACAACTCCAGCTTCTC-3′. The correct gene targeting in all F1 animals was then tested by Southern blot analysis of the tail DNA samples using probes external to the targeting region. The strategy of Southern blot is shown in Supplemental Figure 1C. The following primers for 5′ arm were used. Forward: 5′-AAATCCCCTATGTCACCCCAGGAT-3′ and reverse: 5′-AAACTGCCCACATGTAAGTGCAGC-3′. The following primers for 3′ arm were used. Forward: 5′-GAGATTTCAGGAGCACGATGTGGA-3′ and reverse: 5′-TGGGGTGGTCACCTAAGGTTAGACA-3′.

The miR-150^fl/+^ mice harboring an allele of loxP-flanked miR-150 were bred to generate miR-150^fl/fl^ mice. αMHC-Cre mice (41), in which the expression of Cre recombinase is controlled by the promoter of the cardiac-specific marker gene, were then intercrossed with miR-150^fl/fl^ mice to generate miR-150^fl/+^;αMHC-Cre offspring. The miR-150^fl/+^;αMHC-Cre mice were then crossed back to miR-150^fl/fl^ mice to obtain the miR-150 cKO mice (miR-150^fl/fl^;αMHC-Cre), as shown in Supplemental Figure 1D. Mice were maintained on a C57BL/6J background, and genetically matched Cre-negative miR-150^fl/fl^ mice were used as controls. Genotyping for αMHC was done using primers (5′-ATGACAGACAGATCCCTCCTATCTCC-3′ and 5′-CTCATCACTCGTTGCATCATCGAC-3′) to amplify a 300 bp gene product specific for the transgene gene. Genotyping for miR-150^fl/fl^ was done with the primers of 5′-GAAGGGTTCCTGTCCTTGTTGGC-3′ and 5′-AGTAAGGGTGGAGCCTCTGACCT-3′, resulting in band sizes of 250 bp for the WT allele and 300 bp for the floxed allele.

### Hypomorphic Sprr1a mutant mouse strain.

*Sprr1a*-hypomorphic heterozygous mice (*Sprr1a^hypo/+^*) were obtained from the Mutant Mouse Resource & Research Centers (MMRRC_049856-UCD). This mutant mouse line was generated using the CRISPR/Cas9 strategy. In brief, CRISPR guides and the Cas9 protein were microinjected or electroporated into C57BL/6N zygotes, and progeny were screened for the desired mutation. Founders were mated to C57BL/6N breeders, and derived F1 progeny were identified by PCR and sequencing. F1 mice were then mated again to C57BL/6N breeders to generate F2 mice, which were identified by PCR and sequencing. The CRISPR-mediated genetic alteration resulted in the deletion of 237 bp in exon 2, as well as an amino acid change after residue 44 and early truncation 21 amino acids later. The obtained *Sprr1a^hypo/+^* mice were bred to generate *Sprr1a^hypo/hypo^* mice and WT littermates for the current study. For genotyping, following primers were used (Forward common primer: 5′-TGT GGA AGT CAG CAT GGA G-3′, Reverse WT primer: 5′-CAG GGC TCT GGC CCC TTG-3′, and Reverse mutant primer: 5′-GGA TAG ACA GCA GCC TCA GC-3′).

### Human heart samples.

Samples of failing human hearts were collected from the LVs of ischemic cardiomyopathy and nonischemic cardiomyopathy patients undergoing orthotopic cardiac transplantation, as previously described (42). All patients had been diagnosed with HFrEF. LV tissues were dissected and snap-frozen in liquid nitrogen. The frozen samples were then stored in the specimen storage facility at the Indiana Clinical and Translational Sciences Institute located in the Indiana University School of Medicine. Nonfailing LV tissues were obtained from donor hearts not suitable for transplantation, were collected, and were stored in the same manner. Demographic characteristics of these LV tissue samples are provided in Supplemental Table 1.

### Mouse model of myocardial infarction (MI).

Eight- to 12-week-old miR-150 cKO, miR-150^fl/fl^, *Sprr1a^hypo/hypo^*, or WT mice were subjected to MI as we previously published (35, 43). Briefly, animals were anesthetized using 1%–4% inhalant isoflurane and placed on a heating pad. Animals were intubated and ventilated with oxygen using a PhysioSuite MouseVent ventilator (Kent Scientific). The LAD coronary artery was visualized under a stereoscope and ligated by using an 8-0 nylon suture. Regional ischemia was confirmed by visual inspection for discoloration of the occluded distal myocardium. Sham-operated animals underwent the same procedure without LAD occlusion. One dose of buprenorphine SR Lab (0.05 mg/kg; ZooPharm) was given s.c. immediately before the surgery. We used responses to toe/skin pinch and heart rate for the optimal anesthesia and appropriate postoperative monitoring plan.

### Transthoracic echocardiography.

Left ventricular performance was examined by 2-dimensional echocardiography using a Vevo 2100 Ultrasound (Visual Sonics) before surgery and after MI (3 days and 2, 4, and 8 weeks) as previously published (35, 43). We used M-mode tracings to measure anterior and posterior wall thicknesses at end diastole and end systole. The following parameters were also obtained: left ventricular internal diameter (LVID) in either diastole (LVIDd) or systole (LVIDs), EDV, and ESV. A single operator blinded to mouse genotypes performed echocardiography and data analysis. FS was calculated according to the following formula: FS (%) = ([LVIDd – LVIDs]/LVIDd) × 100. EF was calculated by: EF (%) = ([EDV-ESV]/EDV) × 100. All other LV performance parameters were also obtained as shown in Supplemental Tables 2–5 and Supplemental Tables 7–9.

### Fractionation of cardiac cell types from mouse hearts.

The fractionation of highly purified cardiac cell populations was performed as previously published, with some modifications (14). In brief, freshly harvested mouse hearts were dissociated with a gentleMACS Dissociator (Miltenyi Biotec) according to the manufacturer’s instructions. The dissociated cells were subsequently processed for cell separation. An incubation step with CD31 antibodies coupled to microbeads was performed and subjected to magnetic affinity cell sorting according to the manufacturer’s recommendations (mouse CD31 microbead endothelial isolation kit; Miltenyi Biotec). An additional incubation step with CD31^–^ fraction and CD45 antibodies coupled to microbeads was performed and subjected to magnetic affinity cell sorting (mouse CD45 microbead leukocyte isolation kit; Miltenyi Biotec). CMs and cardiac fibroblasts (CFs) were then separated by a sedimentation step as described (44).

### Statistics.

Data are presented as mean ± SEM from independent experiments with different biological samples per group. The following statistical tests were used: unpaired 2-tailed *t* test for comparisons between 2 groups, 1-way ANOVA with Tukey multiple-comparison test for multiple groups, 2-way ANOVA with Tukey multiple-comparison test for comparisons between 2 groups with different treatments, and 2-way repeated-measures ANOVA with Bonferroni post hoc test for 2 groups over time. Unpaired 2-tailed *t* test was based on assumed normal distributions. *P* < 0.05 was considered statistically significant. *P* values are indicated as follows: * or ^#^*P* < 0.05, ** or ^##^*P* < 0.01, and *** or ^###^*P* < 0.001.

### Study approval.

The use of animals in this study was conformed to the *Guide for the Care and Use of Laboratory Animals* (National Academies Press, 2011). Mice were euthanized by thoracotomy under 1%–4% inhalant isoflurane. All experiments with mice were performed according to the protocols approved by the IACUC at the Indiana University (the approval reference 19018). Eight- to 12-week-old C57BL/6J mice of both sexes were used. All of the procedures involving human samples were conformed to the principles of the Declaration of Helsinki and approved by the Indiana University IRB (the approval reference 08-018). Written informed consent was received from all participants prior to inclusion in the study.

## Author contributions

TA, BM, SK, NPB, MNS, and IK designed research studies, directed the study, and wrote the manuscript. TA, BM, SK, NPB, and MNS conducted most of the experiments, acquired the data, analyzed the data, and prepared the figures. IK supervised the study and provided financial support. CC, YT, SL, RMP, and MSW helped to analyze the data and to write the manuscript.

## Supplementary Material

Supplemental data

Supplemental table 6

## Version 1 08/17/2021

In-Press Preview

## Version 2 09/22/2021

Electronic publication
