# Supplementary material for: Cardiomyocyte microRNA-150 confers cardiac protection and directly represses proapoptotic small proline–rich protein 1A
Source: JCI Insight. 2021 Sep 22;6(18):e150405. doi: 10.1172/jci.insight.150405 (PMC8492334; doi:10.1172/jci.insight.150405)
Supplement: Supplemental data [file jciinsight-6-150405-s203.pdf]

Supplemental data

Supplemental methods

Mouse study

C57BL/6 mice of both genders were used for the current study. Animal research was performed according to animal welfare regulations of the IACUC Committee at Indiana University. All procedures were conducted to conform with NIH guidelines. Carvedilol (Carv; Sigma-Aldrich) was dissolved in DMSO and mini-osmotic pumps (Alzet model 2001; DURECT Corporation) were then used to deliver Carv at the rate of 19 mg/kg/day for 7 days. In control animals, 10% DMSO was administrated as a vehicle. Left ventricle (LV) tissues were then snap-frozen in the liquid nitrogen as we published (1, 2).

Histopathological and immunohistochemical analyses

Morphometric analyses of heart size and weights were conducted as we published (3-5). Histopathological analyses of heart tissues such as fibrosis via Masson's trichrome staining were performed as described (6, 7). For gross histological assessment, heart sections were stained with hematoxylin and eosin (H&E). Cardiac sections were also stained for TUNEL to assess apoptosis via DNA fragmentation using *In Situ* Cell Death Detection Kit (Sigma-Aldrich) as we published (8). Briefly, DNA fragments were labeled with fluorescein-conjugated dUTP using terminal deoxynucleotidyl transferase. The total number of nuclei was determined with DAPI-stained blue nuclei. All TUNEL-positive green nuclei were then counted. Digital photographs of fluorescence were obtained with a Keyence microscope (BZ-X810) and processed with Adobe Photoshop CC 2021. Moreover, cleaved-caspase 3 staining was conducted using the Dako Autostainer Link 48. In brief, cardiac sections were de-paraffinized, rehydrated, as well as subjected to 5 minutes of an EDTA antigen retrieval in a pressure cooker, 15 minutes of endogenous enzyme block, 60 minutes of primary antibody incubation (cleaved caspase-3, rabbit polyclonal [1:200 dilution, 9661, Cell Signaling]), and 30 minutes of Dako EnVision-HRP reagent

incubation. Signals were detected by adding substrate hydrogen peroxide using diaminobenzidine (DAB) as a chromogen followed by hematoxylin counterstaining. Stained (brown) cells were quantified as number of positive cells X 100/total cell infiltrates in 6 random microscopic (40X) fields in each slice.

Transcriptome analysis

Total RNAs from 12 independent mouse LVs (3 sham miR-150$^{fl/fl}$, 3 sham miR-150 cKO, 3 MI miR-150$^{fl/fl}$ and 3 MI miR-150 cKO) were prepared as described (9). RNA quantity and quality were assessed by the Synergy LX FA Multi-Mode Microplate Reader (BioTek Instruments). The DNA microarray experiments were performed using the Mouse Microarray version 3.0 (8 x 60K, Arraystar Inc) designed for the global profiling of 24,881 protein-coding transcripts as described (9-13).

Labeling and array hybridization

RNA labeling and array hybridization for 12 independent mouse LVs were conducted according to the One-Color Microarray-Based Gene Expression Analysis protocol (Agilent Technology) as described (9, 14). The Quick Amp Labeling Kit (Agilent Technologies) was used for sample labeling, and hybridization was conducted in the SureHyb Hybridization Chamber (Agilent Technologies). In brief, mRNAs were purified by using mRNA-ONLY™ Eukaryotic mRNA Isolation Kit (Epicentre). Each sample was then transcribed into fluorescent cRNAs by using Flash RNA Labeling Kit (Arraystar Inc). Next, the labeled cRNAs were purified by RNeasy Mini Kit (Qiagen). Each labeled cRNA was then fragmented, and the mixture was heated at 60°C for 30min. Lastly, labeled cRNAs were loaded into gasket slides and assembled to microarray slides. The slides were then incubated for 17h at 65°C in a hybridization oven (Agilent Technologies), and scanned by using the DNA Microarray Scanner G2505C (Agilent Technologies).

Analysis of microarray dataset

The Feature Extraction software version 11.0.1.1 (Agilent Technologies) was used to analyze array images as we published (9). Fifteen positive probes for housekeeping genes and 20 negative probes are also included onto the array for hybridization quality control. In addition to these normalization methods, we performed more robust quantile normalization across all 12 samples than normalizing with one/some control probes (*i.e.,* the entire intensity distributions were normalized across the arrays, not just some reference points). The subsequent data processing was then conducted using the GeneSpring GX version 12.1 software package (Agilent Technologies). After quantile normalization of the raw data, we chose mRNAs, in which at least 6 out of 12 samples exhibited flags in Present or Marginal (All Targets Value), for further data analyses. We identified differentially expressed (DE) mRNAs with statistical significance through Volcano Plot filtering between the two compared groups of samples. All these DE mRNAs can be found from the spread sheet in Supplemental Table 6. The fold change is presented as the absolute ratio (no log scale) of the averaged normalized intensities between two groups.

We performed the pathway analysis for DE genes to identify the biological pathway by utilizing the GeneSpring Software GX version 12.1 (Agilent Technologies) and the latest Kyoto Encyclopedia of Genes and Genomes (KEGG; http://www.genome.jp/kegg) database. We used Fisher's exact test to calculate the *P* values of the Pathway ID and converted to enrichment score by negative log10 transformation.

Cell culture and transfection

Mouse adult atrial cardiomyocyte (CM) HL-1 cells and rat embryonic ventricular CM H9c2 cells were used as we published (6, 8). CMs were transfected with an Ambion Silencer™ Negative Control siRNA (Life Technologies, cat#: AM4611) or siRNAs targeting rat *Sprr1a* (Dharmacon, 499660) using Lipofectamine™ 3000 reagent (Invitrogen) as previously described (2, 5). For loss-of-function studies, we transfected Ambion Anti-miR™ specific to miR-150 (MH10070; Life

Technologies) or a miR inhibitor negative control (4464076; Life Technologies) as we published (5). Moreover, we transfected miR-150 mimics (MC10070; Life Technologies) for gain-of-function studies. CMs were stimulated with Carv (1 μM; Sigma-Aldrich) in serum-free media for 24h as we published (8).

Simulated ischemia reperfusion (sI/R) assays

CMs were transfected with miR inhibitors or siRNAs and incubated in an ischemia buffer. CMs were then placed in the hypoxic chamber (5% CO_2, 0.1% O_2) for 3h followed by 4h of reperfusion-mimicking conditions (*i.e.,* replacing the ischemic buffer with normoxic cell medium) as previously described (5, 15). CMs were then processed for quantitative real-time RT-PCR, TUNEL staining and Western blotting.

Quantitative real-time RT-PCR

Total RNAs were prepared using Trizol Reagent (Invitrogen) and treated with RNase-free DNase I (Invitrogen) as we published (16, 17). To measure mature miR-150, the TaqMan MicroRNA Reverse Transcription Kit (Life Technologies) was used to generate cDNAs. We used the miR-150 Taqman probe (000473; Life Technologies) to measure both human and rodent mature miRs by Real-Time RT-PCR. U6 snRNA probe, 001973 (Life Technologies) is used for an endogenous control. cDNAs for genes were generated using SuperScript III reverse transcriptase (Invitrogen) and random hexamers. Gene expression was detected using Taqman Gene expression assays for mouse (*Acta1*, Mm00808218_g1; *Ctgf*, Mm01192933_g1; *Col1a1,* Mm00801666_g1; *Tnfa*, Mm00443258_m1; *Il6*, Mm00446190_m1; *p53*, Mm01731290_g1; *P2x7r*, Mm00440578_m1; *Tnnt2*, Mm01290256_m1; *Ddr2*, Mm00445615_m1; *Pecam1*, Mm01242584_m1; *Ptprc*, Mm01293577_m1; *Ank1*, Mm00482889_m1; *Comp*, Mm00489490_m1; *Sprr1a*, Mm01962902_s1 and *Gapdh*, Mm99999915_g1 for an endogenous control), rat (*Sprr1a*, Rn02061965_s1; *Klf13*, Rn01477773_m1; *Ing4*,

Rn01185205_m1 and *Gapdh*, Rn01775763_g1 for an endogenous control), and human (*SPRR1A*, Hs00954595_s1 and *GAPDH*, Hs02786624_g1 for an endogenous control). Quantitative real time RT-PCR reactions were analyzed using a QuantStudio 3 Detection System (Life Technologies) as we published (17). Expression compared to endogenous controls was calculated using $2^{-\Delta\Delta Ct}$ and expression levels were normalized to control.

Western blotting and antibodies

Hearts and cardiomyocytes were washed once with PBS and solubilized in Nonidet P-40 lysis buffer as previously described (4). Lysate samples were resolved by SDS-PAGE and transferred to PVDF (Bio-Rad) for immunoblotting. SPRR1A (ab125374, rabbit, Abcam), EGR2 (AV100880, rabbit, Sigma-Aldrich), β-ACTIN (A5441, mouse, Sigma-Aldrich) and GAPDH (sc-47724, mouse, Santa Cruz) primary antibodies were purchased and used at dilutions of 1:1,000 each. Detection was carried out using ECL (Amersham Biosciences).

Luciferase reporter-based miR targeting assay

The human *SPRR1A* region with the miR-150 binding site was cloned into the pmirGLO Dual-Luciferase miR Target Expression Vector (E1130, Promega). The following oligonucleotide pairs were designed, annealed, and ligated into the pmirGLO Vector to generate WT and mutant luciferase constructs: WT forward primer, 5'-AAACTAGCGGCCGCTAGTAGGGCTGTGCACTTT***TGGGAG***AT-3', WT reverse primer, 5'-CTAGAT***CTCCCA***AAAGTGCACAGCCCTACTAGCGGCCGCTAGTTT-3', mutant forward primer, 5'-AAACTAGCGGCCGCTAGTAGGGCTGTGCACTTT***TTAAG***AT-3', and mutant reverse primer, 5'-CTAGAT***CTTAA***AAAGTGCACAGCCCTACTAGCGGCCGCTAGTTT-3'. Inserts and insertion sites were confirmed by ~140bp insert when digested with NotI due to NotI sites in the vector and oligonucleotides, followed by sequencing for all plasmids. We transfected H9c2 cells in 96-well plates with 0.1 μg of one of the luciferase plasmids along with either 50nM of miR mimic

control or miR-150 mimic. At 72h after transfection, we collected cells for luciferase activities in Firefly and Renilla buffers that are measured by Dual-Glo Luciferase Assay System (E2920, Promega) with the Synergy LX FA Multi-Mode Microplate Reader (BioTek Instruments) as described (4, 5).

Supplemental references

1. Kim IM, et al. beta-arrestin1-biased beta1-adrenergic receptor signaling regulates microRNA processing. *Circ Res.* 2014;114(5):833-44.
2. Kim IM, et al. Beta-blockers alprenolol and carvedilol stimulate beta-arrestin-mediated EGFR transactivation. *Proc Natl Acad Sci U S A.* 2008;105(38):14555-60.
3. Bayoumi AS, et al. A carvedilol-responsive microRNA, miR-125b-5p protects the heart from acute myocardial infarction by repressing pro-apoptotic bak1 and klf13 in cardiomyocytes. *J Mol Cell Cardiol.* 2017;114:72-82.
4. Bayoumi AS, et al. MicroRNA-532 protects the heart in acute myocardial infarction, and represses prss23, a positive regulator of endothelial-to-mesenchymal transition. *Cardiovasc Res.* 2017;113(13):1603-14.
5. Tang Y, et al. MicroRNA-150 protects the mouse heart from ischaemic injury by regulating cell death. *Cardiovasc Res.* 2015;106(3):387-97.
6. Ramakrishna S, et al. Myocardium defects and ventricular hypoplasia in mice homozygous null for the Forkhead Box M1 transcription factor. *Dev Dyn.* 2007;236(4):1000-13.
7. Kim IM, et al. The Forkhead Box m1 transcription factor stimulates the proliferation of tumor cells during development of lung cancer. *Cancer Res.* 2006;66(4):2153-61.
8. Park KM, et al. Carvedilol-responsive microRNAs, miR-199a-3p and -214 protect cardiomyocytes from simulated ischemia-reperfusion injury. *Am J Physiol Heart Circ Physiol.* 2016;311(2):H371-83.
9. Teoh JP, et al. Identification of gene signatures regulated by carvedilol in mouse heart. *Physiol Genomics.* 2015;47(9):376-85.
10. Zhu H, et al. miR-17-92 cluster promotes cholangiocarcinoma growth: evidence for PTEN as downstream target and IL-6/Stat3 as upstream activator. *Am J Pathol.* 2014;184(10):2828-39.
11. Cossette SM, et al. Sucrose non-fermenting related kinase enzyme is essential for cardiac metabolism. *Biol Open.* 2014;4(1):48-61.
12. Liao JM, et al. Global effect of inauhzin on human p53-responsive transcriptome. *PLoS ONE.* 2012;7(12):e52172.
13. Stueckle TA, et al. Chronic occupational exposure to arsenic induces carcinogenic gene signaling networks and neoplastic transformation in human lung epithelial cells. *Toxicol Appl Pharmacol.* 2012;261(2):204-16.
14. Patterson TA, et al. Performance comparison of one-color and two-color platforms within the MicroArray Quality Control (MAQC) project. *Nat Biotechnol.* 2006;24(9):1140-50.
15. Aurora AB, et al. MicroRNA-214 protects the mouse heart from ischemic injury by controlling Ca(2)(+) overload and cell death. *J Clin Invest.* 2012;122(4):1222-32.
16. Kim IM, et al. The forkhead box m1 transcription factor is essential for embryonic development of pulmonary vasculature. *J Biol Chem.* 2005;280(23):22278-86.
17. Kim IM, et al. Gene deletion screen for cardiomyopathy in adult Drosophila identifies a new notch ligand. *Circ Res.* 2010;106(7):1233-43.

Supplemental Figure 1

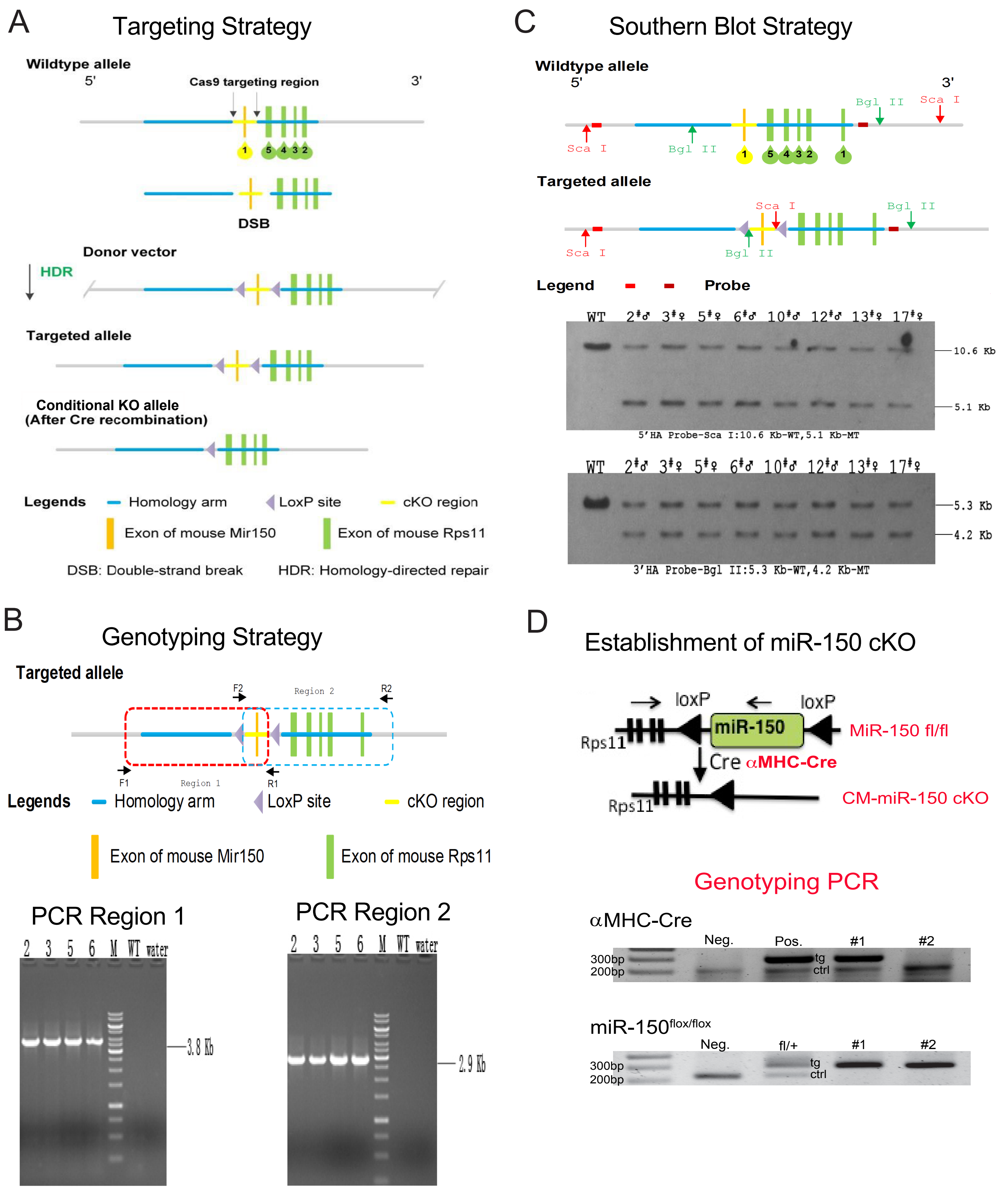

Supplemental Figure 1. Generation of a novel cardiac-specific miR-150 knockout mouse line. A, Targeting strategy of cardiac-specific knockout (KO) of miR-150 in vivo. **B-C**, Genotyping and Southern blot strategies to screen F1 miR-150$^{fl/+}$ mice. Genotyping PCR (**B**) and Southern blot (**C**) images show germline transmission of the targeted miR-150 floxed allele. **D**, Establishment of the cardiomyocyte (CM)-specific miR-150 conditional KO (cKO) mouse model. **Top**, Targeting scheme and mouse crossing. **Bottom**, Representative genotyping results of miR-150$^{fl/fl}$ and αMHC-Cre mice. Target (tg) and control (ctrl) bands are shown. Neg.= negative control and Pos.= positive control.

Supplemental Figure 2

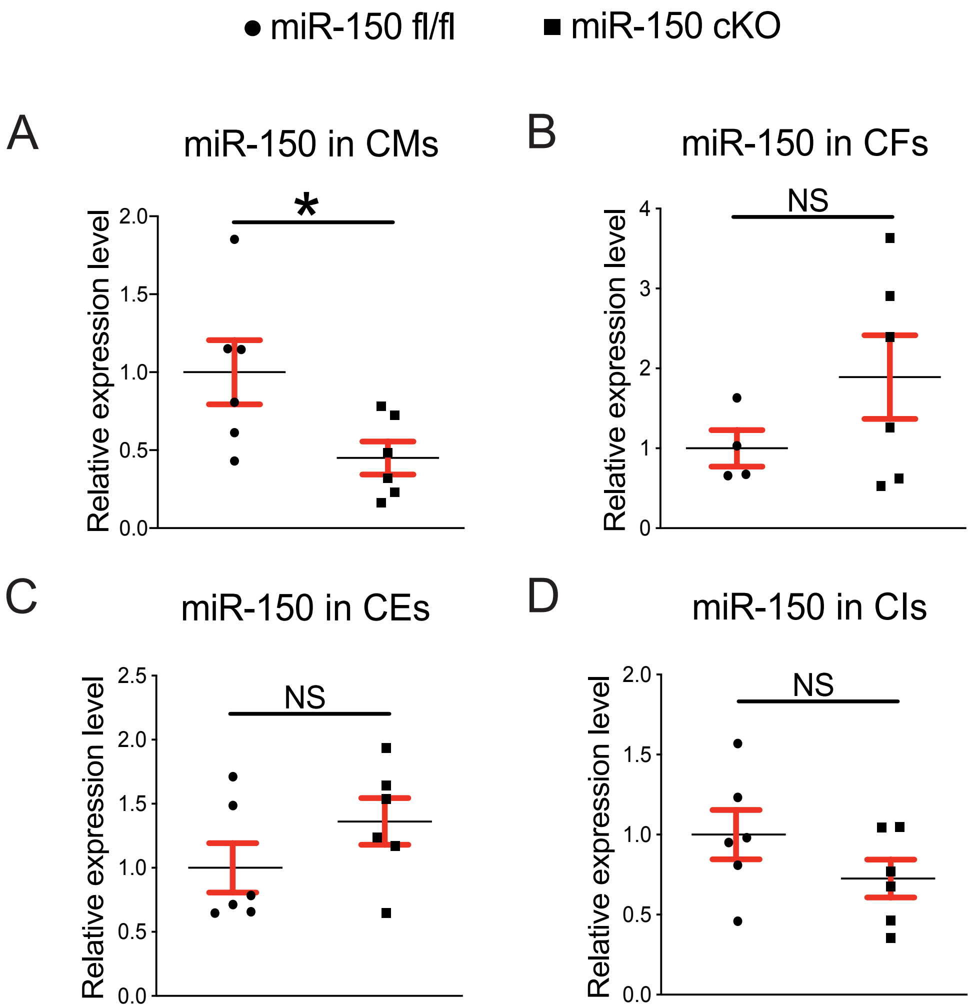

Supplemental Figure 2. Confirmation of cardiomyocyte-specific miR-150 conditional knockout. A, QRT-PCR analyses of miR-150 in CMs isolated from miR-150$^{fl/fl}$ or miR-150 cKO mice. Unpaired 2-tailed t-test. **P* < 0.05 vs. CMs from miR-150$^{fl/fl}$. **B-D,** QRT-PCR analyses of miR-150 in cardiac fibroblasts [CFs] (**B**), cardiac endothelial cells [CEs] (**C**) and cardiac inflammatory cells [CIs] (**D**) isolated from miR-150$^{fl/fl}$ or miR-150 cKO mice. NS: not significant. Data are presented as mean ± SEM.

Supplemental Figure 3

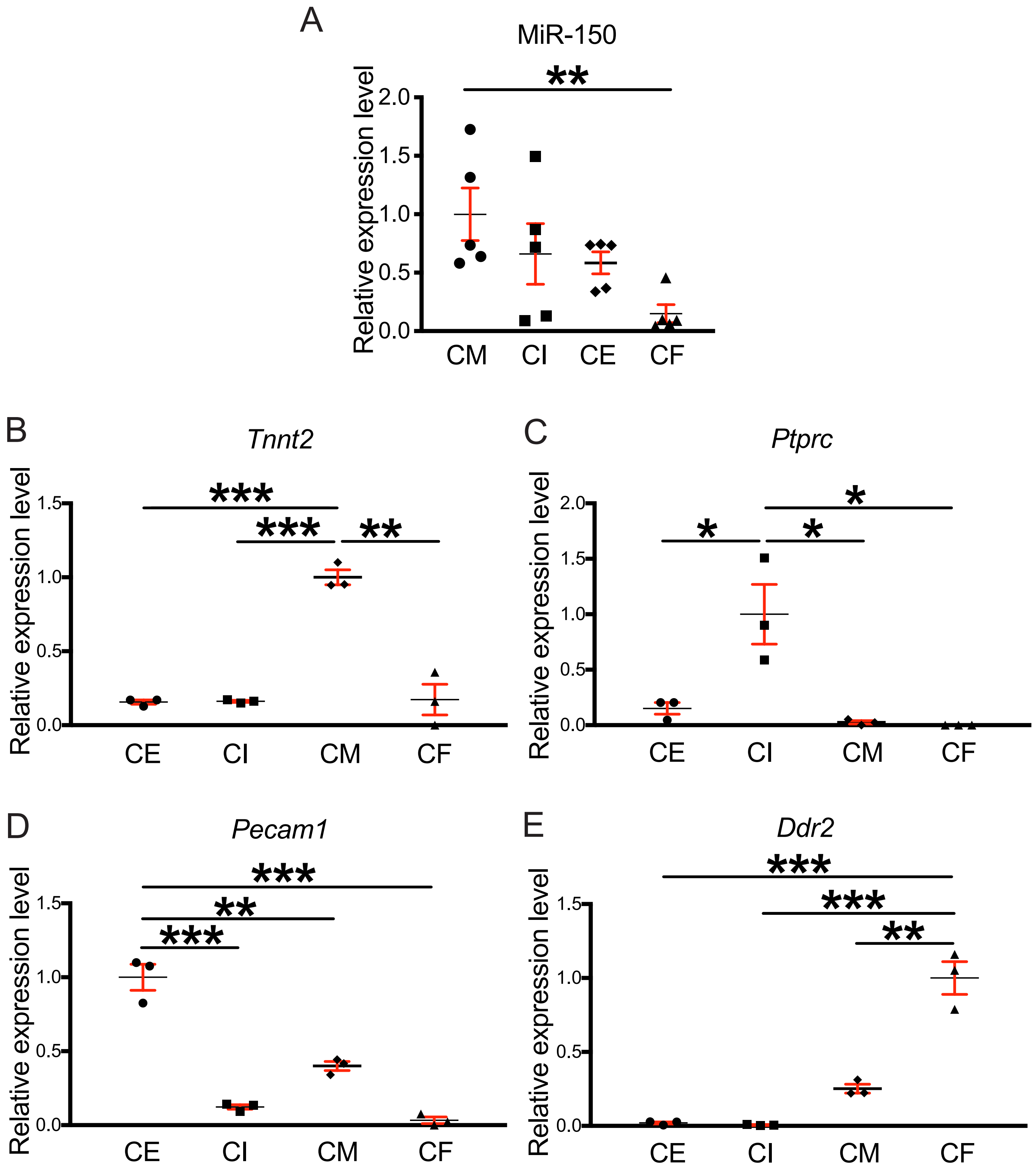

Supplemental Figure 3. The expression patterns of miR-150 in distinct myocardial cells. A, QRT-PCR analyses of miR-150 in myocardial cells (CMs, CIs, CEs and CFs) isolated from WT adult mouse hearts. One-way ANOVA with Tukey multiple comparison test. $^{**}P < 0.01$ vs. CM. **B-E**, The expression of cell type-specific markers was detected by QRT-PCR. *Tnnt2*: cardiac muscle troponin T (**B**). *Ptprc*: protein tyrosine phosphatase receptor type C (**C**). *Pecam1*: platelet endothelial cell adhesion molecule-1 (**D**). *Ddr2*: discoidin domain receptor 2 (**E**). One-way ANOVA with Tukey multiple comparison test. $^{*}P<0.05$, $^{**}P<0.01$ or $^{***}P<0.001$ vs. CM (**B**), CI (**C**), CE (**D**) or CF (**E**). Data are presented as mean ± SEM.

Supplemental Figure 4

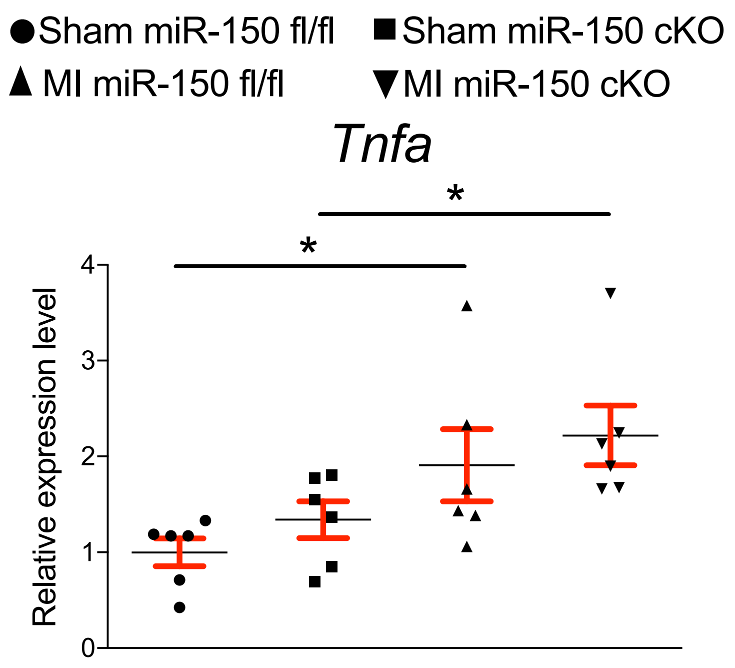

Supplemental Figure 4. MiR-150 cKO MI hearts exhibit normal expression of pro-inflammatory *Tnfa*. QRT-PCR analyses of *Tnfa* in miR-150 cKO hearts compared to miR-$150^{fl/fl}$ controls at post-MI 4 weeks. Data are represented as fold change of gene expression normalized to *Gapdh*. One-way ANOVA with Tukey multiple comparison test. *P<0.05 vs. sham. Data are presented as mean ± SEM.

Supplemental Figure 5

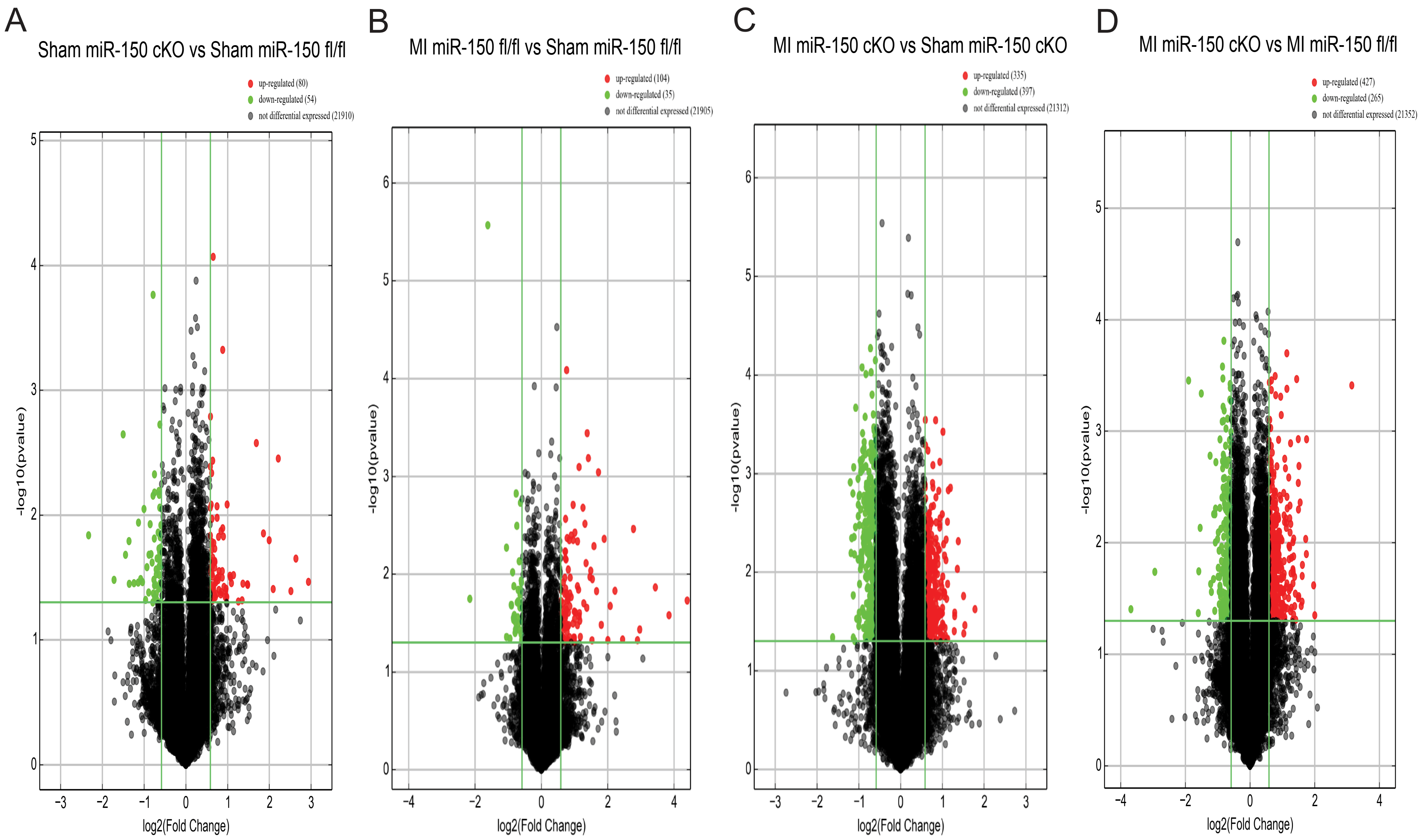

Supplemental Figure 5. Volcano plot analyses of differentially expressed cardiac genes to discover gene signatures regulated by miR-150 selectively in cardiomyocytes. A-D, Volcano Plots are constructed using fold-change values and *P* values that allow visualization of the relationship between fold-change and statistical significance, taking both magnitudes of change and variability into consideration. The vertical lines correspond to 1.5-fold up and down, and the horizontal line represents a *P* value of 0.05. So, the red (up) or green (down) points in the plot represent the differentially expressed (DE) mRNAs with statistical significance. DE genes in sham miR-150 cKO compared to sham miR-$150^{fl/fl}$ controls are shown in **A**, DE genes in MI miR-$150^{fl/fl}$ compared to sham miR-$150^{fl/fl}$ controls are shown in **B**, DE genes in MI miR-150 cKO compared to sham miR-150 cKO are shown in **C**, and DE genes in MI miR-150 cKO compared to MI miR-$150^{fl/fl}$ at 4 weeks post-MI are shown in **D.**

Supplemental Figure 6

Upregulated Signaling Pathways

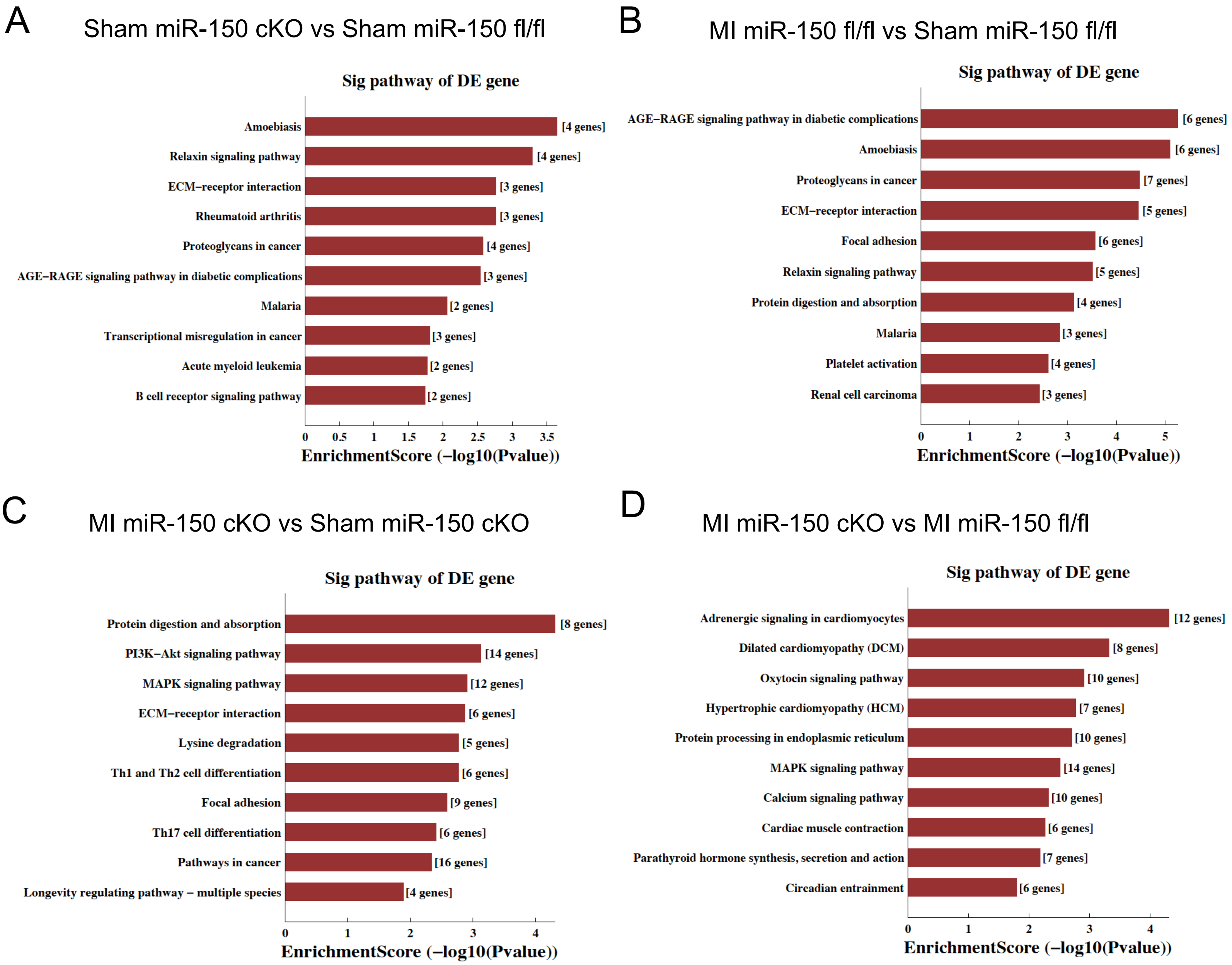

Supplemental Figure 6. Signaling pathway analyses of differentially upregulated genes. The functional pathway analysis is performed by mapping genes to KEGG pathways. Fisher's exact test is used. The *P* value indicates the significance of the Pathway between groups (**A:** sham miR-150 cKO compared to sham miR-$150^{fl/fl}$ control, **B:** MI miR-$150^{fl/fl}$ compared to sham miR-$150^{fl/fl}$, **C:** MI miR-150 cKO compared to sham miR-150 cKO, and **D:** MI miR-150 cKO compared to MI miR-$150^{fl/fl}$). Enrichment Score is the value of the Pathway ID, which equals to -log10(P value). The bar plots represent the top ten enrichment score values of the significant enrichment pathway. Among differentially expressed (DE) genes discovered in microarray analyses (Supplemental Table 6), upregulated genes are only shown.

Supplemental Figure 7

Downregulated Signaling Pathways

A MI miR-150 fl/fl vs Sham miR-150 fl/fl

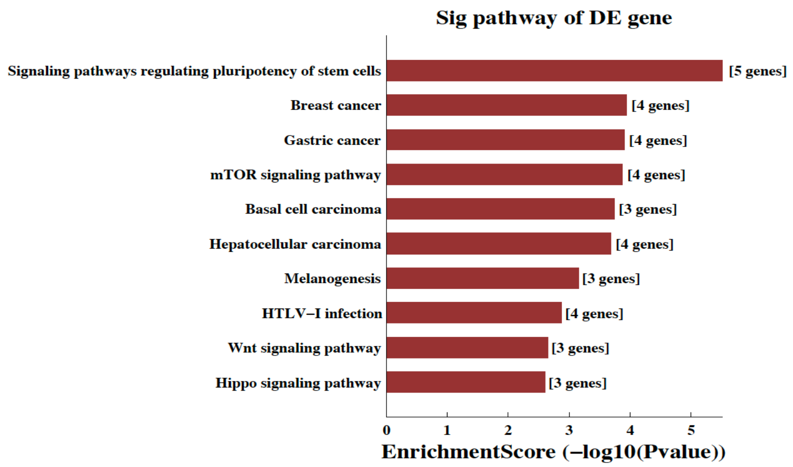

B MI miR-150 cKO vs Sham miR-150 cKO

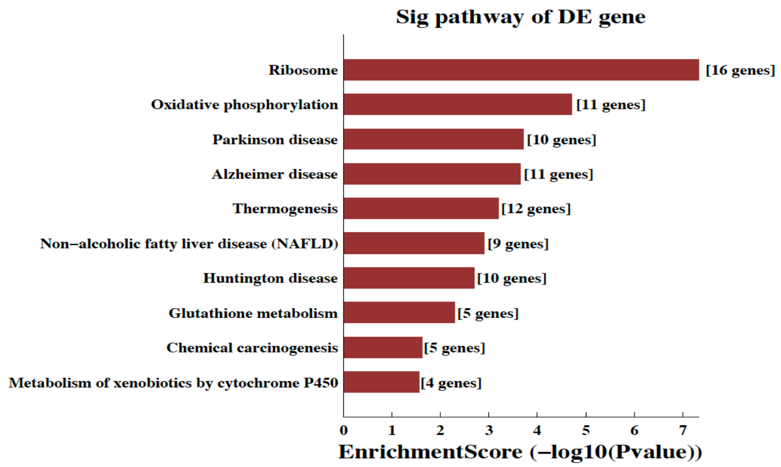

C MI miR-150 cKO vs MI miR-150 fl/fl

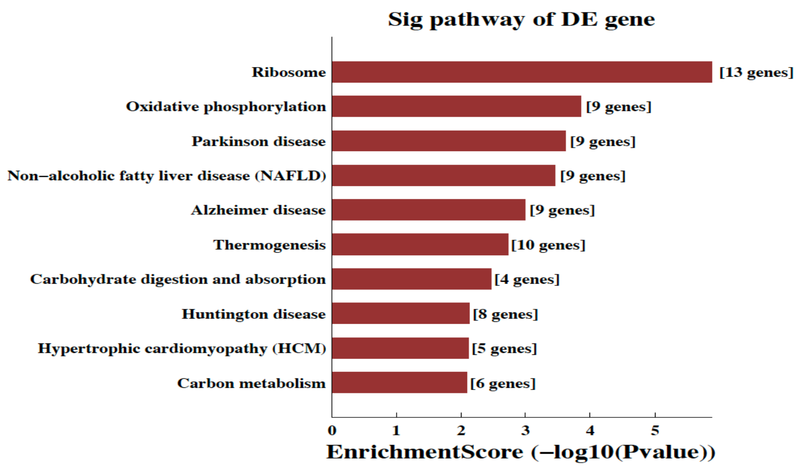

Supplemental Figure 7. Signaling pathway analyses of differentially downregulated genes. The functional pathway analysis is performed by mapping genes to KEGG pathways. Fisher's exact test is used. The *P* value indicates the significance of the Pathway between groups (**A:** MI miR-150$^{fl/fl}$ compared to sham miR-150$^{fl/fl}$, **B:** MI miR-150 cKO compared to sham miR-150 cKO, and **C:** MI miR-150 cKO compared to MI miR-150$^{fl/fl}$). Enrichment Score is the value of the Pathway ID, which equals to -log10(P value). The bar plots represent the top ten enrichment score values of the significant enrichment pathway. Among differentially expressed (DE) genes discovered in microarray analyses (Supplemental Table 6), downregulated genes are only shown.

Supplemental Figure 8

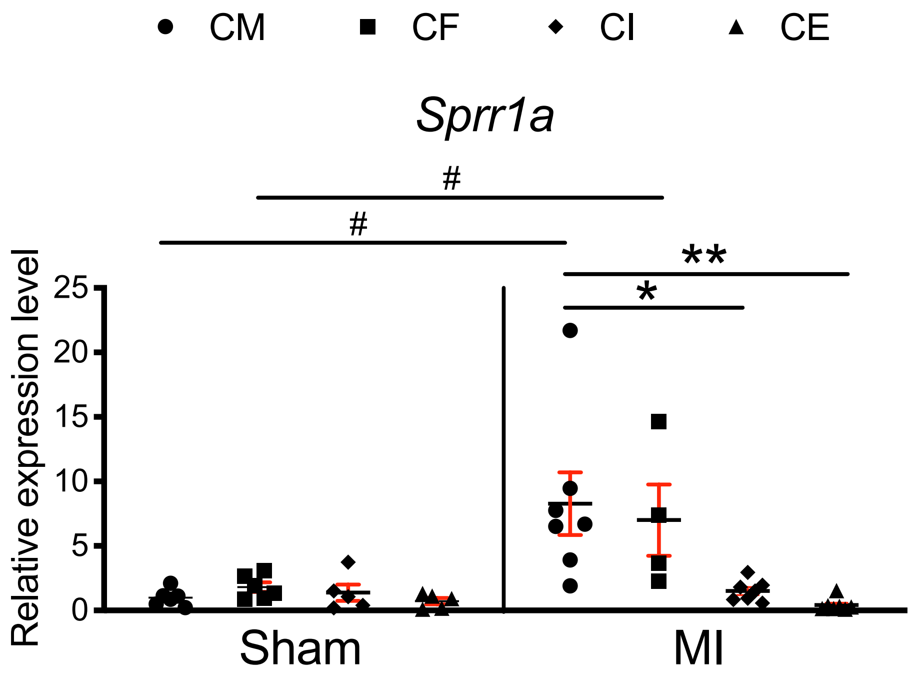

Supplemental Figure 8. *Sprr1a* is ubiquitously expressed in myocardial cells, and is upregulated in CMs and CFs isolated from ischemic myocardium. QRT-PCR expression analysis of *Sprr1a* in cardiomyocytes (CMs), cardiac fibroblasts (CFs), cardiac inflammatory cells (CIs), and cardiac endothelial cells (CEs) isolated from adult mouse heart at 7 days post-MI. N=4-7. One-way ANOVA with Tukey multiple comparison test. $^{*}P<0.05$ or $^{**}P<0.01$ vs. CM. $^{\#}P<0.05$ vs. sham. Data are presented as mean ± SEM.

Supplemental Figure 9

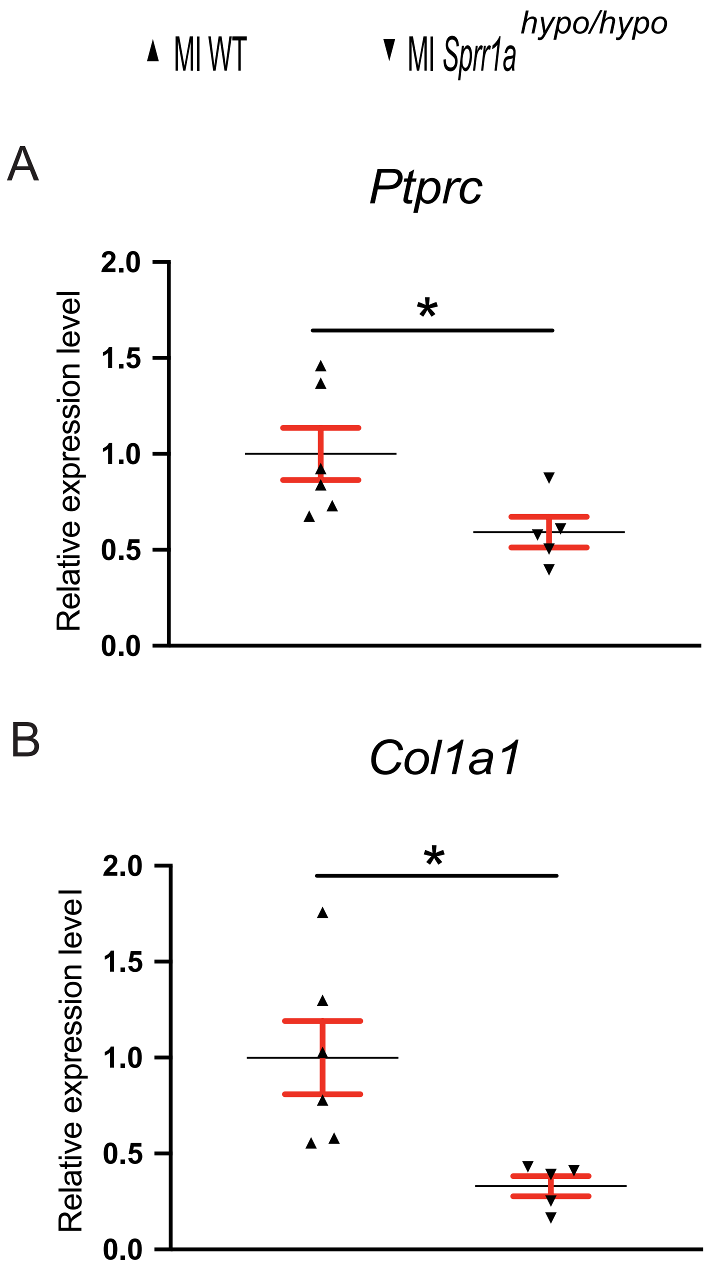

Supplemental Figure 9. Downregulation of *Sprr1a* in mice reduces cardiac expression of inflammatory *Ptprc* and fibrotic *Col1a1* after MI. QRT-PCR analysis of inflammatory *Ptprc* (**A**) and fibrotic *Col1a1* (**B**) expression in *Sprr1a*$^{hypo/hypo}$ hearts compared to WT controls at 8 weeks post-MI. N=5-6 per group. Data are shown as fold induction of gene expression normalized to *Gapdh*. Unpaired 2-tailed t-test. *P<0.05 vs. MI WT. Data are presented as mean ± SEM.

Supplemental Figure 10

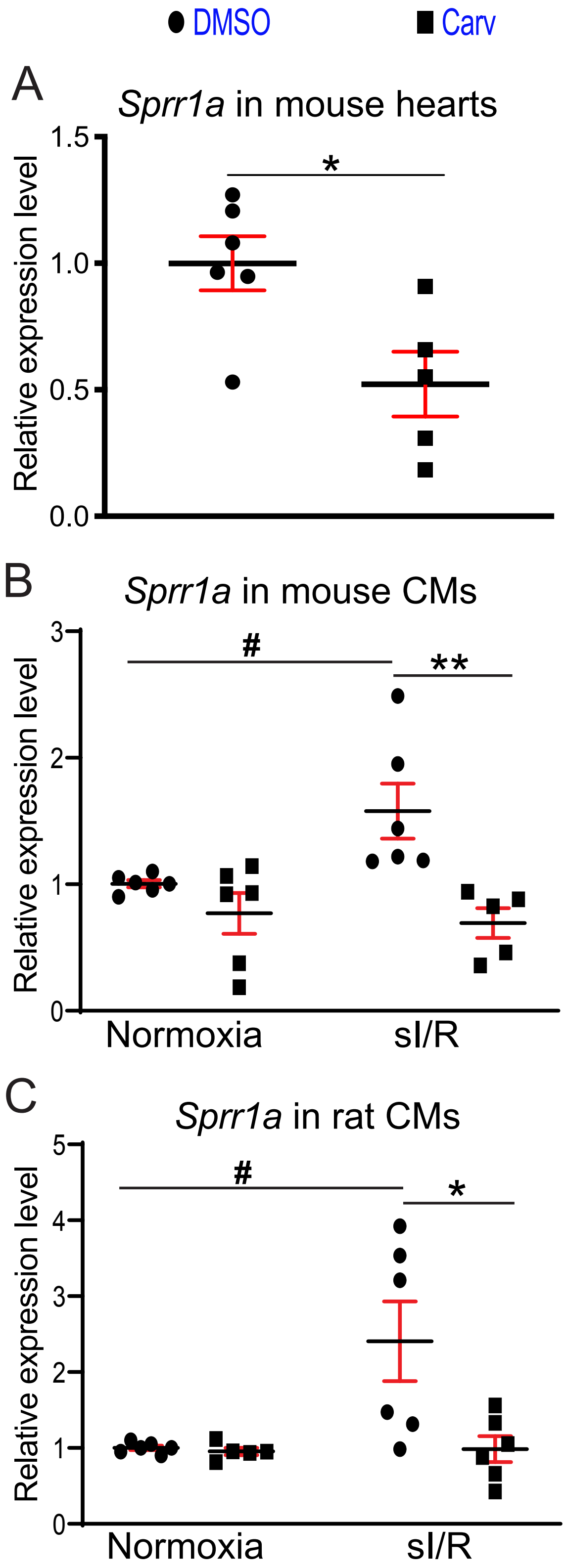

Supplemental Figure 10. Carvedilol represses *Sprr1a* in mouse hearts or cardiomyocytes. **A,** *Sprr1a* was measured by QRT-PCR in adult mouse hearts treated with carvedilol (Carv) or vehicle for 7 days. Carv inhibits *Sprr1a* in mouse hearts. **B-C**, Cardiomyocytes were stimulated with 1 μM Carv for 24h and subjected to either normoxia or sI/R. QRT-PCR analysis for *Sprr1a* was then conducted. Carv inhibits *Sprr1a* in HL-1 (**B**) and H9c2 (**C**) cells only subjected to sI/R. Moreover, *Sprr1a* is upregulated after sI/R. Unpaired 2-tailed t-test (**A**). One-way ANOVA with Tukey multiple comparison test (**B-C**). **P*<0.05 or ***P*<0.01 vs. DMSO. #*P*<0.05 vs. basal DMSO. Data are presented as mean ± SEM.

Supplemental Figure 11

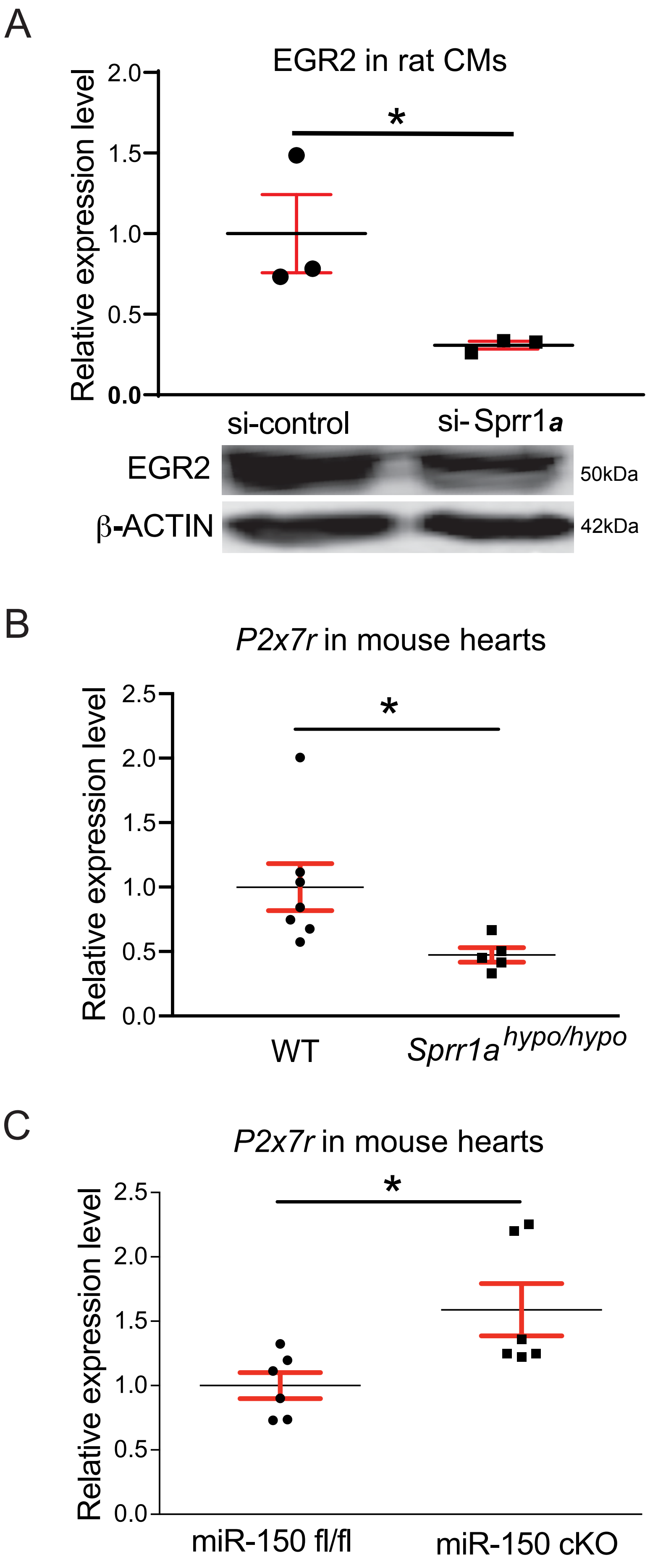

Supplemental Figure 11. Knockdown of cardiomyocyte *Sprr1a* reduces pro-apoptotic EGR2 expression, as well as *Sprr1a* knockdown in mice decreases the cardiac expression of pro-apoptotic and a known miR-150 target *P2x7r*, concurrent with upregulation of *P2x7r* in miR-150 cKO mouse hearts. A, H9c2 cells were transfected with control scramble siRNA (si-control) or *Sprr1a* siRNA (si-*Sprr1a*). Western Blotting analysis of EGR2 was performed. N=3 per group. Data are shown as fold induction of protein expression normalized to β-ACTIN. Unpaired 2-tailed t-test. **P*<0.05 vs. si-control. **B-C**, QRT-PCR analysis of *P2x7r* expression in *Sprr1a*$^{hypo/hypo}$ hearts compared to WT controls (**B**) or miR-150 cKO hearts compared to miR-150$^{fl/fl}$ controls (**C**). N=5-7 per group. Data are shown as fold induction of gene expression normalized to *Gapdh*. Unpaired 2-tailed t-test. **P*<0.05 vs. WT or miR-150$^{fl/fl}$. Data are presented as mean ± SEM.

Supplemental Figure 12

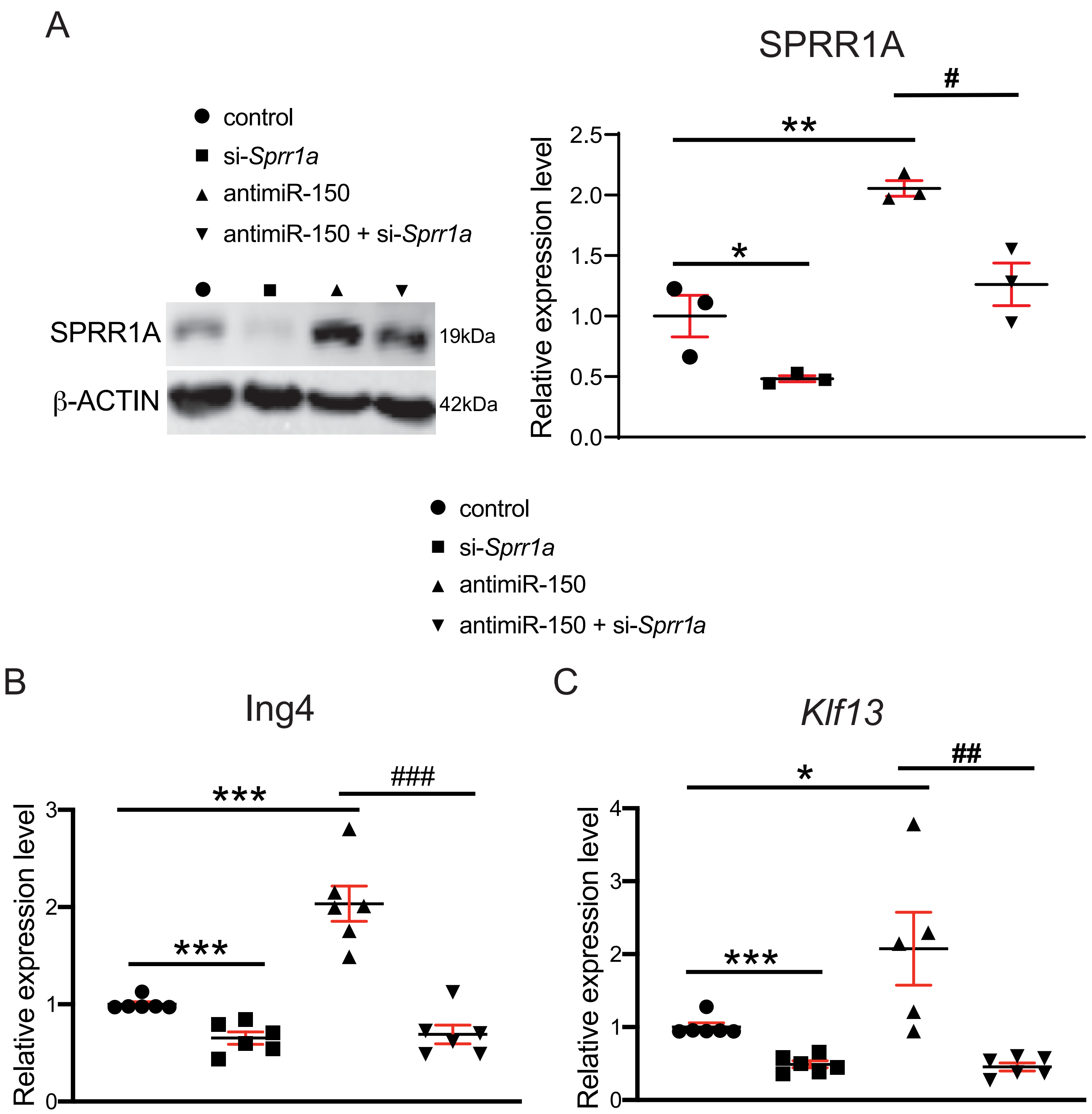

Supplemental Figure 12. *Sprr1a* is necessary for miR-150-dependent regulation of pro-apoptotic *Ing4* or *Klf13* expression in cardiomyocytes. H9c2 cells were transfected with control scramble siRNA (si-control) or *Sprr1a* siRNA (si-*Sprr1a*) and with antimiR scramble control or antimiR-150. Western Blotting analysis of SPRR1A (**A**) and QRT-PCR analysis for pro-apoptotic *Ing4* (**B**) or *Klf13* (**C**) were performed. Knockdown of *Sprr1a* reduces pro-apoptotic *Ing4* or *Klf13* expression in cardiomyocytes. RNA interference with *Sprr1a* in cardiomyocytes also prevents increased expression of pro-apoptotic *Ing4* or *Klf13* mediated by antimiR-150 treatment. N=3-6 per group. Data are shown as fold induction of or protein expression normalized to β-ACTIN (**A**) or gene expression normalized to *Gapdh* (**B-C**). One-way ANOVA with Tukey multiple comparison test. *P<0.05, **P<0.01 or ***P<0.001 vs. control: either si-control or antimiR control. $^{\#}P$<0.05, $^{\#\#}P$<0.01 or $^{\#\#\#}P$<0.001 vs. antimiR-150. Data are presented as mean ± SEM.

Supplemental Table 1. Demographic characteristics of human LV tissue samples

**Etiology**	**Non-failing**	**HFrEF**
Number	7	12
Age (years)	77.14 ± 5.21	52.50 ± 5.70
Sex male (%)	57.14	66.67
LVEF (%)	62 ± 0.03	22 ± 2.00

Supplemental Table 2. Echocardiographic parameters in anesthetized miR-150 fl/fl or miR-150 cKO mice before they were randomly assigned to 4 experimental groups.

Day 0	MiR-150 fl/fl		MiR-150 cKO	
	Sham (n=13)	MI (n=12)	Sham (n=13)	MI (n=14)
CO (ml/min)	19.65 ± 0.88	19.27 ± 0.89	19.76 ± 1.05	18.70 ± 0.64
EF (%)	75.23 ± 0.81	74.65 ± 0.75	75.33 ± 0.70	74.58 ± 0.69
FS (%)	43.12 ± 0.71	42.59 ± 0.66	43.26 ± 0.64	42.48 ± 0.60
HR (bpm)	550 ± 12.48	538 ± 12.93	540 ± 12.94	539 ± 11.60
SV (µl)	35.82 ± 1.59	35.80 ± 1.30	36.53 ± 1.58	34.69 ± 0.89
Volume, diastole (µl)	47.67 ± 2.13	47.95 ± 1.64	48.46 ± 1.98	46.53 ± 1.16
Volume, systole (µl)	11.85 ± 0.71	12.15 ± 0.54	11.93 ± 0.54	11.84 ± 0.45
LVAW, diastole (mm)	0.73 ± 0.02	0.74 ± 0.02	0.74 ± 0.02	0.75 ± 0.01
LVAW, systole (mm)	1.10 ± 0.04	1.12 ± 0.04	1.08 ± 0.03	1.09 ± 0.03
LVID, diastole (mm)	3.40 ± 0.06	3.41 ± 0.05	3.42 ± 0.06	3.37 ± 0.03
LVID, systole (mm)	1.93 ± 0.05	1.96 ± 0.03	1.94 ± 0.04	1.94 ± 0.03
LVPW, diastole (mm)	0.82 ± 0.02	0.83 ± 0.02	0.84 ± 0.03	0.79 ± 0.03
LVPW, systole (mm)	1.12 ± 0.03	1.13 ± 0.03	1.17 ± 0.03	1.12 ± 0.03

Abbreviations: MI = myocardial infarction, CO = cardiac output, EF = ejection fraction, FS = fractional shortening, HR = heart rate, SV = stroke volume, LVAW = left ventricular anterior wall thickness, LVID = left ventricular interior diameter, LVPW = left ventricular posterior wall thickness. All values are expressed as mean ± SEM.

Supplemental Table 3. Echocardiographic parameters in anesthetized miR-150 fl/fl or miR-150 cKO mice at 3 days after Sham or MI surgery.

Day 3 post-surgery	MiR-150 fl/fl		MiR-150 cKO	
	Sham (n=13)	MI (n=12)	Sham (n=13)	MI (n=14)
CO (ml/min)	19.59 ± 0.67	15.19 ± 0.61***	17.72 ± 0.83	13.43 ± 0.88**
EF (%)	73.88 ± 0.93	54.89 ± 1.31***	73.98 ± 0.88	48.93 ± 1.26***##
FS (%)	41.98 ± 0.79	27.87 ± 0.80***	41.92 ± 0.78	24.12 ± 0.74***##
HR (bpm)	550 ± 8.50	536 ± 10.82	550 ± 13.28	540 ± 10.73
SV (µl)	35.68 ± 1.21	28.43 ± 1.18***	32.37 ± 1.54	24.86 ± 1.58**
Volume, diastole (µl)	48.41 ± 1.86	52.32 ± 3.05	43.71 ± 1.91	51.38 ± 3.72
Volume, systole (µl)	12.73 ± 0.83	23.90 ± 2.09***	11.34 ± 0.58	26.52 ± 2.26***
LVAW, diastole (mm)	0.78 ± 0.02	0.75 ± 0.02	0.77 ± 0.02	0.74 ± 0.02
LVAW, systole (mm)	1.14 ± 0.03	1.00 ± 0.05*	1.11 ± 0.03	0.92 ± 0.03***
LVID, diastole (mm)	3.42 ± 0.05	3.53 ± 0.08	3.28 ± 0.06	3.49 ± 0.10
LVID, systole (mm)	1.99 ± 0.05	2.55 ± 0.08***	1.90 ± 0.04	2.65 ± 0.09***
LVPW, diastole (mm)	0.80 ± 0.02	0.80 ± 0.02	0.79 ± 0.03	0.76 ± 0.03
LVPW, systole (mm)	1.04 ± 0.03	1.02 ± 0.03	1.05 ± 0.02	0.95 ± 0.03*

Abbreviations: MI = myocardial infarction, CO = cardiac output, EF = ejection fraction, FS = fractional shortening, HR = heart rate, SV = stroke volume, LVAW = left ventricular anterior wall thickness, LVID = left ventricular interior diameter, LVPW = left ventricular posterior wall thickness. All values are expressed as mean ± SEM. All values are expressed as mean ± SEM. $^{*}P$<0.05, $^{**}P$<0.01 or $^{***}P$<0.001 vs. sham within same group. $^{\#\#}P$<0.01 vs. miR-150 fl/fl MI. Only parameters, which were statistically significant between groups, are highlighted with red fonts.

Supplemental Table 4. Echocardiographic parameters in anesthetized miR-150 fl/fl or miR-150 cKO mice at 2 weeks after Sham or MI surgery.

2 weeks post-surgery	MiR-150 fl/fl		MiR-150 cKO	
	Sham (n=13)	MI (n=12)	Sham (n=13)	MI (n=13)
CO (ml/min)	19.17 ± 0.58	16.68 ± 0.73*	20.18 ± 0.67	17.92 ± 0.85*
EF (%)	75.23 ± 0.69	49.93 ± 1.82***	73.88 ± 0.72	44.02 ± 1.67***#
FS (%)	43.03 ± 0.63	24.99 ± 1.08***	42.05 ± 0.63	21.59 ± 0.94***#
HR (bpm)	552 ± 11.29	548 ± 11.82	553 ± 10.25	541 ± 11.11
SV (μl)	34.82 ± 1.09	30.55 ± 1.36*	36.58 ± 1.21	33.13 ± 1.54
Volume, diastole (μl)	46.28 ± 1.36	61.55 ± 2.39***	49.30 ± 1.44	76.56 ± 4.62***##
Volume, systole (μl)	11.46 ± 0.45	31.00 ± 1.97***	12.71 ± 0.46	43.42 ± 3.68***##
LVAW, diastole (mm)	0.75 ± 0.02	0.69 ± 0.02*	0.73 ± 0.02	0.60 ± 0.03**#
LVAW, systole (mm)	1.17 ± 0.03	0.87 ± 0.05***	1.19 ± 0.03	0.80 ± 0.05***
LVID, diastole (mm)	3.36 ± 0.04	3.78 ± 0.06***	3.47 ± 0.05	4.13 ± 0.10***##
LVID, systole (mm)	1.92 ± 0.03	2.84 ± 0.07***	1.98 ± 0.02	3.25 ± 0.11***##
LVPW, diastole (mm)	0.83 ± 0.03	0.76 ± 0.02	0.84 ± 0.03	0.75 ± 0.02**
LVPW, systole (mm)	1.18 ± 0.05	0.99 ± 0.02**	1.19 ± 0.02	1.00 ± 0.04***

Abbreviations: MI = myocardial infarction, CO = cardiac output, EF = ejection fraction, FS = fractional shortening, HR = heart rate, SV = stroke volume, LVAW = left ventricular anterior wall thickness, LVID = left ventricular interior diameter, LVPW = left ventricular posterior wall thickness. All values are expressed as mean ± SEM. All values are expressed as mean ± SEM. $^{*}P<0.05$, $^{**}P<0.01$ or $^{***}P<0.001$ vs. sham within same group. $^{\#}P<0.05$ or $^{\#\#}P<0.01$ vs. miR-150 fl/fl MI. Only parameters, which were statistically significant between groups, are highlighted with red fonts.

Supplemental Table 5. Echocardiographic parameters in anesthetized miR-150 fl/fl or miR-150 cKO mice at 4 weeks after sham or MI surgery.

4 weeks post-surgery	MiR-150 fl/fl		MiR-150 cKO	
Echocardiography	Sham (n=12)	MI (n=12)	Sham (n=12)	MI (n=12)
CO (ml/min)	21.63 ± 1.03	18.84 ± 1.30	21.10 ± 0.85	20.29 ±1.66
EF (%)	73.02 ± 1.01	49.63 ± 1.72***	71.80 ± 0.50	43.60 ± 1.67***#
FS (%)	41.35 ± 0.85	24.82 ± 1.00***	40.17 ± 0.43	21.34 ± 0.93***#
HR (bpm)	553 ± 11.27	547 ± 11.18	571 ± 14.52	543 ± 10.53
SV (µl)	39.02 ± 1.35	34.58 ± 2.49	36.96 ± 1.08	35.17 ± 1.85
Volume, diastole (µl)	53.72 ± 2.37	69.78 ± 6.45*	51.50 ± 1.54	83.46 ± 5.31***
Volume, systole (µl)	14.69 ± 1.10	35.20 ± 4.75***	14.54 ± 0.55	48.59 ± 4.08***#
LVAW, diastole (mm)	0.80 ± 0.02	0.63 ± 0.03***	0.75 ± 0.02	0.55 ± 0.02***#
LVAW, systole (mm)	1.17 ± 0.04	0.83 ± 0.07***	1.18 ± 0.03	0.75 ± 0.04***
LVID, diastole (mm)	3.57 ± 0.07	3.96 ± 0.14*	3.51 ± 0.04	4.31 ± 0.11***
LVID, systole (mm)	2.10 ± 0.06	2.95 ± 0.14***	2.10 ± 0.03	3.40 ± 0.11***#
LVPW, diastole (mm)	0.84 ± 0.03	0.79 ± 0.04	0.88 ± 0.02	0.71 ± 0.04***
LVPW, systole (mm)	1.18 ± 0.03	1.04 ± 0.05*	1.17 ± 0.04	0.98 ± 0.03**
Morphometric data	Sham (n=6)	MI (n=6)	Sham (n=6)	MI (n=6)
LVW/BW (mg/g)	3.35 ± 0.03	3.53 ± 0.05*	3.48 ± 0.04#	3.70 ± 0.05**#

Abbreviations: MI = myocardial infarction, CO = cardiac output, EF = ejection fraction, FS = fractional shortening, HR = heart rate, SV = stroke volume, LVAW = left ventricular anterior wall thickness, LVID = left ventricular interior diameter, LVPW = left ventricular posterior wall thickness, HW = heart weight, BW = body weight, LVW = left ventricular weight. All values are expressed as mean ± SEM. All values are expressed as mean ± SEM. $^{*}P$<0.05, $^{**}P$<0.01 or $^{***}P$<0.001 vs. sham within same group. $^{\#}P$<0.05 vs. miR-150 fl/fl MI. Only parameters, which were statistically significant between groups, are highlighted with red fonts.

Supplemental Table 6. Differentially expressed genes.

Please refer to the Supplemental Table 6_Differentially Expressed Genes.xls file

Supplemental Table 7. Echocardiographic parameters in anesthetized WT or *Sprr1a*$^{hypo/hypo}$ mice before they were randomly assigned to 4 experimental groups.

Day 0	WT		*Sprr1a*$^{hypo/hypo}$	
	Sham (n=18)	MI (n=18)	Sham (n=18)	MI (n=17)
CO (ml/min)	21.19 ± 0.88	20.27 ± 0.73	21.85 ± 0.51	20.32 ± 0.65
EF (%)	72.44 ± 0.80	73.08 ± 0.70	73.48 ± 0.84	71.52 ± 0.72
FS (%)	40.79 ± 0.67	41.27 ± 0.60	41.74 ± 0.72	39.93 ± 0.57
HR (bpm)	536 ± 11.87	537 ± 9.59	541 ± 10.14	555 ± 10.82
SV (µl)	39.67 ± 1.68	37.73 ± 1.16	40.56 ± 1.09	36.70 ± 1.09
Volume, diastole (µl)	53.67 ± 1.76	51.66 ± 1.58	55.32 ± 1.58	51.48 ± 1.76
Volume, systole (µl)	14.83 ± 0.74	13.92 ± 0.59	14.76 ± 0.72	14.78 ± 0.78
LVAW, diastole (mm)	0.80 ± 0.02	0.79 ± 0.02	0.79 ± 0.01	0.75 ± 0.01
LVAW, systole (mm)	1.22 ± 0.03	1.20 ± 0.04	1.26 ± 0.03	1.18 ± 0.03
LVID, diastole (mm)	3.57 ± 0.05	3.52 ± 0.04	3.62 ± 0.04	3.51 ± 0.05
LVID, systole (mm)	2.12 ± 0.04	2.07 ± 0.03	2.11 ± 0.04	2.11 ± 0.04
LVPW, diastole (mm)	0.83 ± 0.01	0.81 ± 0.02	0.80 ± 0.02	0.78 ± 0.02
LVPW, systole (mm)	1.27 ± 0.02	1.23 ± 0.02	1.26 ± 0.03	1.21 ± 0.03

Abbreviations: MI = myocardial infarction, CO = cardiac output, EF = ejection fraction, FS = fractional shortening, HR = heart rate, SV = stroke volume, LVAW = left ventricular anterior wall thickness, LVID = left ventricular interior diameter, LVPW = left ventricular posterior wall thickness. All values are expressed as mean ± SEM.

Supplemental Table 8. Echocardiographic parameters in anesthetized WT or *Sprr1a*$^{hypo/hypo}$ mice at 4 weeks after Sham or MI surgery.

4 weeks post-surgery	WT		*Sprr1a*$^{hypo/hypo}$	
	Sham (n=18)	MI (n=18)	Sham (n=18)	MI (n=17)
CO (ml/min)	20.57 ± 0.73	16.77 ± 0.90**	21.58 ± 0.99	19.17 ± 0.73#
EF (%)	70.77 ± 1.31	46.43 ± 1.23***	71.90 ± 0.93	50.16 ± 1.4***
FS (%)	39.52 ± 0.98	22.90 ± 0.72***	40.37 ± 0.77	25.20 ± 0.84***#
HR (bpm)	532 ± 8.43	521 ± 10.94	552 ± 12.88	538 ± 9.10
SV (µl)	38.78 ± 1.40	32.24 ± 1.76**	39.09 ± 1.45	35.56 ± 1.09
Volume, diastole (µl)	55.02 ± 2.01	69.93 ± 4.32**	54.30 ± 1.91	72.47 ± 3.89***
Volume, systole (µl)	16.23 ± 1.17	37.69 ± 2.86***	15.21 ± 0.71	36.91 ± 3.09***
LVAW, diastole (mm)	0.80 ± 0.01	0.74 ± 0.02*	0.81 ± 0.02	0.72 ± 0.02**
LVAW, systole (mm)	1.21 ± 0.03	0.93 ± 0.04***	1.25 ± 0.02	0.91 ± 0.04***
LVID, diastole (mm)	3.61 ± 0.06	3.97 ± 0.10**	3.59 ± 0.06	4.04 ± 0.09***
LVID, systole (mm)	2.18 ± 0.06	3.06 ± 0.09***	2.14 ± 0.04	3.03 ± 0.10***
LVPW, diastole (mm)	0.82 ± 0.01	0.77 ± 0.02*	0.81 ± 0.02	0.77 ± 0.02
LVPW, systole (mm)	1.19 ± 0.02	1.00 ± 0.02***	1.22 ± 0.03	1.03 ± 0.02***

Abbreviations: MI = myocardial infarction, CO = cardiac output, EF = ejection fraction, FS = fractional shortening, HR = heart rate, SV = stroke volume, LVAW = left ventricular anterior wall thickness, LVID = left ventricular interior diameter, LVPW = left ventricular posterior wall thickness. All values are expressed as mean ± SEM. *P<0.05, **P<0.01 or ***P<0.001 vs. sham within same group. #P<0.05 vs. WT MI. Only parameters, which were statistically significant between groups, are highlighted with red fonts.

Supplemental Table 9. Echocardiographic parameters in anesthetized WT or *Sprr1a*$^{hypo/hypo}$ mice at 8 weeks after Sham or MI surgery.

8 weeks post-surgery	WT		*Sprr1a*$^{hypo/hypo}$	
	Sham (n=18)	MI (n=18)	Sham (n=18)	MI (n=17)
CO (ml/min)	21.42 ± 0.82	16.64 ± 0.86***	21.54 ± 0.95	17.35 ± 1.12**
EF (%)	73.54 ± 0.69	44.26 ± 1.32***	71.81 ± 0.61	48.37 ± 1.00***#
FS (%)	41.73 ± 0.57	21.68 ± 0.75***	40.28 ± 0.53	24.01 ± 0.59***#
HR (bpm)	534 ± 10.63	514 ± 7.71	532 ± 11.30	528 ± 11.39
SV (µl)	39.99 ± 1.11	32.60 ± 1.85**	40.40 ± 1.44	32.64 ± 1.78**
Volume, diastole (µl)	54.53 ± 1.73	74.29 ± 4.56***	56.16 ± 1.75	67.98 ± 4.27*
Volume, systole (µl)	14.54 ± 0.74	41.69 ± 3.15***	15.76 ± 0.47	35.34 ± 2.72***
LVAW, diastole (mm)	0.79 ± 0.01	0.73 ± 0.02*	0.76 ± 0.01	0.74 ± 0.02
LVAW, systole (mm)	1.25 ± 0.02	0.92 ± 0.03***	1.22 ± 0.02	0.91 ± 0.04***
LVID, diastole (mm)	3.60 ± 0.05	4.07 ± 0.10***	3.64 ± 0.05	3.93 ± 0.10*
LVID, systole (mm)	2.10 ± 0.04	3.19 ± 0.09***	2.17 ± 0.03	2.99 ± 0.08***
LVPW, diastole (mm)	0.81 ± 0.01	0.74 ± 0.02**	0.79 ± 0.01	0.79 ± 0.01#
LVPW, systole (mm)	1.26 ± 0.02	0.99 ± 0.03***	1.21 ± 0.02	1.08 ± 0.02***#

Abbreviations: MI = myocardial infarction, CO = cardiac output, EF = ejection fraction, FS = fractional shortening, HR = heart rate, SV = stroke volume, LVAW = left ventricular anterior wall thickness, LVID = left ventricular interior diameter, LVPW = left ventricular posterior wall thickness. All values are expressed as mean ± SEM. $^{*}P$<0.05, $^{**}P$<0.01 or $^{***}P$<0.001 vs. sham within same group. $^{\#}P$<0.05 vs. WT MI. Only parameters, which were statistically significant between groups, are highlighted with red fonts.
